# What drives European solidarity? Evidence on identity-based, value-based, and utilitarian explanations

**DOI:** 10.1177/14651165261423107

**Published:** 2026-03-12

**Authors:** Jakob J Eicheler

**Affiliations:** 1 Department of Political Science, 27170 University of Duisburg-Essen, Duisburg, Germany

**Keywords:** European solidarity, European identification, national identification, political orientation, panel data, Germany

## Abstract

This article analyses how and why individuals maintain or change their European solidarity over time using German panel data. I answer two questions: (1) Who maintains their level of European solidarity? (2) Do identity-based, value-based, or utilitarian approaches best explain change in European solidarity? I examine four dimensions of solidarity: territorial, fiscal, and welfare solidarity, and the support for European social citizenship. Despite COVID-19 and Russia's invasion of Ukraine, 44–57% of German respondents maintained stable solidarity. Stability varied little across sociodemographic groups. Those who identified more with Europe and on the economic and transnationalist left were less likely to reduce their solidarity. Transnational political orientation is the main driver of intra-individual change in European solidarity. When individuals come to identify more with Europe, they increase their solidarity, whereas national identification has inconsistent effects. Utilitarian factors, including exposure to regional crises, show little effect on individual solidarity. The findings add nuance to identity-based and utilitarian explanations of how individuals develop and maintain European solidarity.

## Understanding the dynamics of European solidarity

From a theoretical perspective, European solidarity – the individual-level preparedness to share resources with others in the European Union (EU) (Stjernø, 2005: 2) – is fundamental for deeper integration in the EU's social dimension. Although empathy with other Europeans has grown (Clasen, 2023), recent crises had divergent effects on European solidarity: It declined during the COVID-19 pandemic, rose briefly after Russia's invasion of Ukraine, and then returned to previous levels (Russo, 2024). These fluctuations show the need to explain why some individuals maintain their solidarity while others change it.

Existing research points to three main explanations: identity-based, value-based and utilitarian. These approaches focus on national and European identification, political orientation, and material self-interest. However, previous studies use single or repeated cross-sectional data,^1^ which cannot inform on how or why individuals change their solidarity over time. To understand these dynamics, we need to learn how shifts in identification, values, and utilitarian considerations interact with external events in shaping European solidarity.

This article makes both a theoretical and an empirical contribution by being the first study to test whether the three main explanations of European solidarity explain intra-individual change in European solidarity, and not just cross-sectional differences in levels. Using two German panel datasets, the study analyses the drivers of stability and change. Panel data allows to control for time-invariant confounding and reduce omitted-variable bias. This provides a more rigorous test of the three accounts, advancing our understanding of the drivers of European solidarity.

The article finds that, despite numerous crises, 44–57% of respondents maintained (i.e. did not change levels) in European solidarity over months and even years. Stability varies little across sociodemographic groups. Individuals with strong national identification and weak European identification, and those with right-leaning economic and transnational political orientations, were most likely to reduce their solidarity.

The findings highlight value-based factors to most substantially drive change in European solidarity, particularly via shifts in transnational political orientation. Shifts in economic political orientation only affect territorial and fiscal solidarity. National identification shows inconsistent effects, whereas growing European identification is associated with increasing European solidarity. Utilitarian factors have little effect: better perceptions of the national economy are associated only with increasing fiscal solidarity, but personal material conditions and regional crises experiences show no consistent effects.

These results challenge primarily identity-based (Nicoli et al., 2020) and utilitarian (Russo, 2023a: 571) explanations of European solidarity. They emphasise instead the value-driven explanation centred on the transnational cleavage (Hooghe and Marks, 2018). The panel data analysis confirms previous cross-sectional findings on its importance (Kriesi et al., 2024). For an EU constrained by public opinion in its further integration, this article provides insights into when a permissive consensus or constraining dissensus on Social Europe may arise (Hooghe and Marks, 2009).

## Definitions and explanations of stability and change in European solidarity

### The concept of European solidarity

Solidarity is ‘the preparedness to share resources with others by personal contribution to those in struggle or in need and through taxation and redistribution organised by the state’ (Stjernø, 2005: 2) and is tied to an imagined community or group (Lahusen and Grasso, 2018: 5). It can take the form of social, civic, or political solidarity (Scholz, 2008). I focus on civic solidarity – the ‘relationship between citizens within a political state’ (Scholz, 2008: 29) – or in this case, the polity of the EU. ‘European solidarity’ therefore means solidarity with people from other EU countries. Following Reinl and Giebler (2021), identities, political orientations, and socio-economic conditions are expected to shape preferences for European solidarity.

European solidarity is a multidimensional construct, as solidarity itself can take different forms. Gerhards et al. (2019) distinguish fiscal, territorial, and welfare solidarity: ‘(1) [F]iscal solidarity is defined as citizens’ willingness to financially support crisis-affected European countries; (2) territorial solidarity is the willingness to reduce wealth inequalities between EU countries, [… and] (3) welfare solidarity is citizens’ willingness to support Europeans in need […] regardless of where they live in the EU’ (Gerhards et al., 2019: 3).^2^

I extend Gerhards et al.'s (2019) framework to provide a more comprehensive analysis of European solidarity. This article examines a fourth dimension: European social citizenship, defined as ‘granting […] social rights to EU citizens or a Europeanization of social rights’ (Baute et al., 2018: 356–358). This dimension closely relates to opposition to welfare chauvinism directed at EU citizens (Hjorth, 2016). It differs from welfare solidarity in scope: European social citizenship concerns granting social rights to EU citizens within one's own country, whereas welfare solidarity refers to supporting EU citizens in other member states. Table 1 summarises the four dimensions of European solidarity used here and relates them to existing conceptualisations of dimensions of European solidarity by Gerhards et al. (2019), Reinl (2022) and Baute et al. (2018).

**Table 1.** The four dimensions of European solidarity and related concepts in existing conceptualisations.

Dimension	Defined as willingness to …	Gerhards et al. (2019)	Baute et al. (2018)	Reinl (2022)
European social citizenship	Grant national social rights to EU citizens	–	European Social Citizenship	–
Welfare solidarity	Support Europeans in need abroad	Welfare Solidarity	Interpersonal European solidarity	Redistributive transnational solidarity
Territorial solidarity	Reduce wealth inequalities between EU countries	Territorial solidarity		
Fiscal solidarity	Financially support crisis-affected European countries	Fiscal solidarity	Member-state solidarity	Risk-sharing transnational solidarity

### Explaining stability in European solidarity

No study has so far provided a theoretical framework to understand how stable attitudes toward European solidarity are. Seminal work by Converse (1964) and Zaller (1992), as well as recent empirical studies, help identify who is most likely to hold stable views on European solidarity. Zaller's model (Zaller, 1992: 42–51) suggests that those with high political awareness and strongly held opinions are most resistant to change their attitudes.

The ‘impressionable years’ hypothesis (Alwin and Krosnick, 1991) expects younger people to have less stable attitudes because they are still evolving their perceptions and evaluations of the political world. Panel evidence suggests that indeed, older individuals’ attitudes are more stable, as well as the attitudes of the socio-economically advantaged (Milojev et al., 2015). A Dutch study, however, reports greater stability in younger individuals, as well as those higher-educated, male, and with extreme or right-wing views (Zwicker et al., 2020). Similarly, Dutch and US citizens with more strongly held attitudes showed more stability (Turner-Zwinkels and Brandt, 2023).

I therefore expect younger, higher-educated and higher-income individuals, as well as those with initially stronger attitudes, to have more stable attitudes on European solidarity. This includes a stronger identification with Europe and Germany, and more extreme economic and transnational orientations as well as extreme positions on European solidarity itself. Finally, I expect economically right-wing and anti-transnationalist orientations to be associated with higher levels of stability.

### Explaining change in European solidarity

**Figure 1.**
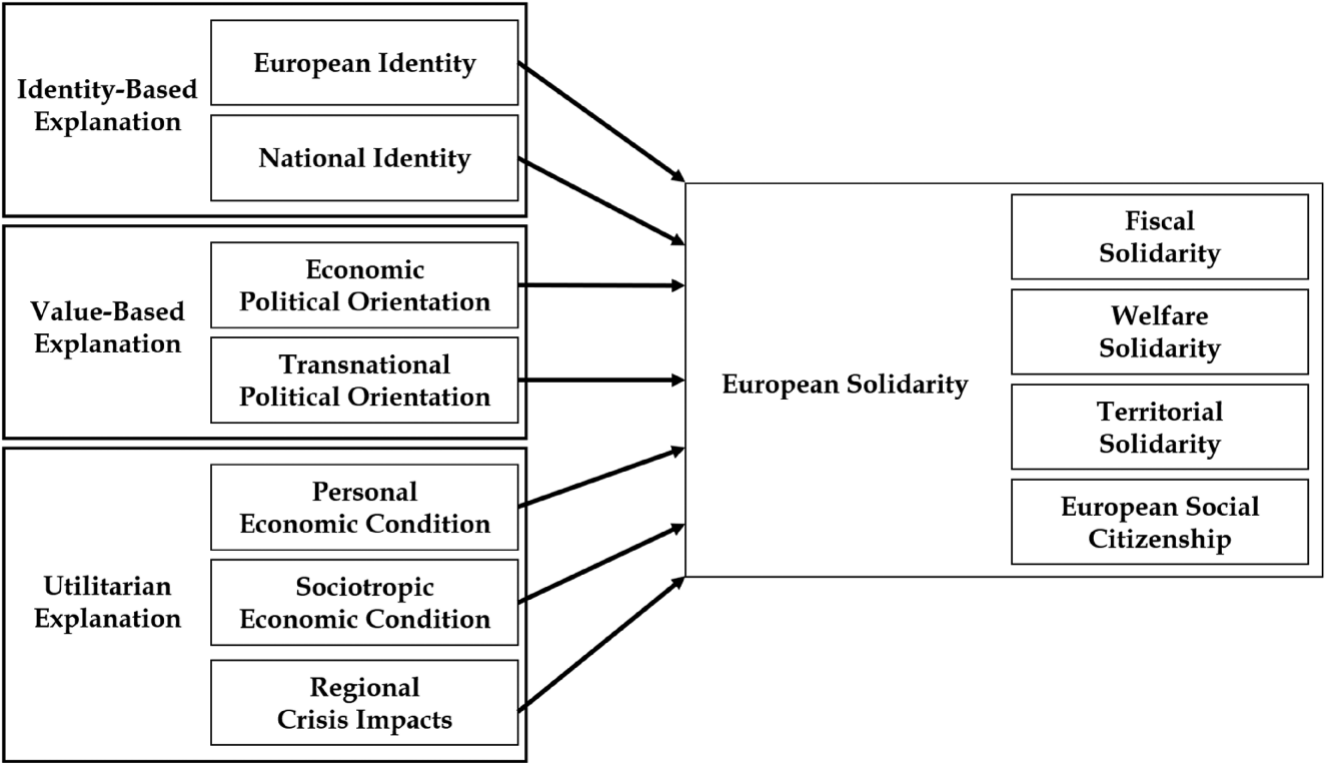
Theoretical model to explain change in European solidarity*.*

What explains change in European solidarity? Similar to support for European integration, these explanations can be organised into identity-based, value-based, utilitarian and cue-taking/benchmarking explanations (Hobolt and De Vries, 2016: 414; Leuffen et al., 2022: 150).^3^ Given the single-country context of Germany in the available panel data, this article cannot address cue-taking or benchmarking. Figure 1 presents the theoretical model, which is similar to models in recent cross-sectional studies of European solidarity (Ciornei and Hernandez Sanchez, 2024; Kriesi et al., 2024; Oana et al., 2025: 9–10; Russo, 2023a). For each explanation, I discuss the mechanism, review previous research, and then derive hypotheses.

#### Identity-based explanation: National and European identity

Social Identity Theory (Tajfel and Turner, 1979) helps explain why people show European solidarity. When individuals feel they identify with and belong to a group, they tend to cooperate with and support its members. They also develop more positive attitudes and behaviours towards perceived in-group members (Ben-Ner et al., 2009). The Common Ingroup Identity Model (Gaertner et al., 2016) adds that individuals can have multiple, nested group affiliations, such as a national identity embedded within a European identity. Identification with Europe is malleable, such as in response to crises (Nicoli et al., 2024) or through the use of political symbols (Bruter, 2009).

When people identify more strongly with Europe and less with their nation, they may shift group boundaries: other Europeans move from being perceived as an out-group to being an in-group. Given that people usually perceive and treat in-group members more positively and pro-socially, such shifts can increase European solidarity by shifting in-group demarcations.

Previous research robustly finds that individuals who identify more with Europe than with their own nation show higher levels of European solidarity (Baute and Pellegata, 2023; Ciornei and Recchi, 2017; Gerhards et al., 2019; Kleider and Stoeckel, 2019; Kuhn et al., 2018; Kyriazi et al., 2023; Russo, 2023a; Vasilopoulou and Talving, 2020; Verhaegen, 2018). Based on these mechanisms and previous research, I test the following hypotheses:

*H1a*: Over time, respondents who identified less strongly with Germany on average increased in European solidarity.

*H1b*: Over time, respondents who identified more strongly with Europe on average increased in European solidarity.

#### Value-based explanation: Economic and transnational political orientation

A value-based approach explains European solidarity through individuals’ economic and transnational political orientation (Kriesi et al., 2008). Both dimensions structure the European political space (Jackson and Jolly, 2021; Johnston and Ollerenshaw, 2020), and can shape European solidarity over time.

The economic left–right dimension – rooted in the cleavage between labour and capital (Lipset and Rokkan, 1967) – has historically structured party systems and political attitudes in Europe. Individuals' placement on this dimension stems largely from how much inequality they accept and how much they resist social change (Jost, 2021: 30). Since European solidarity redistributes resources across member states and individuals, and implies further integration, left-leaning individuals should support it more strongly.

At the individual level, left–right orientation causally affects support for redistribution and for the welfare state (Jæger, 2006, 2008: 363–364). This link is likely to extend beyond the national level to the European level (Kleider and Stoeckel, 2019: 7). When citizens move to the economic left over time, I therefore expect their support for European solidarity to increase. Previous research confirms the link between an economically left-leaning political orientation and European solidarity (Baute et al., 2019; Baute and Pellegata, 2023; Gerhards et al., 2016, 2019; Kuhn et al., 2018; Kyriazi et al., 2023; Russo, 2023a).

A transnational cleavage has emerged across Europe, organised around immigration and European integration (Hooghe and Marks, 2018: 109; Kriesi et al., 2008). Nationalist positions are associated with opposition to European integration, especially in redistributive policy implied by European solidarity (Börzel and Risse, 2020). When citizens shift towards a more transnationalist orientation, I expect European solidarity to increase.

Existing evidence supports this expectation. Kleider and Stoeckel (2019) show that transnational orientation – attitudes towards immigration, same-sex marriage, and privacy rights – strongly predict fiscal solidarity. Visconti and Pellegata (2025) find that transnational orientations have the greatest impact in northern and western EU member states. Several other studies have found that those who support EU membership tend to show greater European solidarity (Baute et al., 2019; Kleider and Stoeckel, 2019; Kuhn et al., 2018; Russo, 2023a). Based on these mechanisms and previous research, I test the following hypotheses:

*H2a*: Over time, respondents who moved to the left in their economic political orientation on average increased in European solidarity.

*H2b*: Over time, respondents who moved to a more transnationalist position in their political orientation on average increased in European solidarity.

#### Utilitarian explanation: Personal and sociotropic material self-interest and crisis impacts

A utilitarian perspective explains European solidarity through personal and sociotropic material self-interest, dependent on perceived costs and benefits. In Germany, perceived benefits of European solidarity are likely to remain fairly stable, even when people perceive their personal or the sociotropic material conditions to worsen. Germany is the largest net contributor to EU funds (Bechtel et al., 2014), and few citizens expect to receive transfers from other member states. But when Germans perceive material conditions to worsen, they may see solidarity as more costly, because contributions appear to reduce national welfare capacity. If costs rise while benefits remain constant, the net utility of European solidarity falls, leading to lower support.

Evidence for the personal material self-interest explanation is mixed. Some studies find that higher social class is associated with greater European solidarity (Kleider and Stoeckel, 2019; Kuhn et al., 2018), while economic insecurity does not appear to be related (Baute et al., 2019; Russo, 2023a). Several studies report higher-income individuals to be more supportive of European solidarity (Bechtel et al., 2014; Kyriazi et al., 2023; Verhaegen, 2018), whereas Gerhards et al. (2019), in a survey across 13 EU countries, find null or even negative associations.

The evidence on the sociotropic material self-interest explanation is equally mixed. Individuals living in more prosperous countries tend to show lower European solidarity (Lengfeld et al., 2015; Russo, 2023a), whereas those who assess the national economy positively tend to show greater European solidarity (Vasilopoulou and Talving, 2020; Verhaegen, 2018). Higher GDP is associated with greater solidarity (Kleider and Stoeckel, 2019; Kuhn et al., 2018; Lengfeld et al., 2015; Vasilopoulou and Talving, 2020), but eurozone residents tend to support it less (Kleider and Stoeckel, 2019; Kuhn et al., 2018). The limited panel data evidence is mixed: Bobzien and Kalleitner (2021) find no link between European solidarity and the belief that Austria benefits from EU support. Goldberg et al. (2021) show Dutch respondents to lower their level of solidarity when perceptions of the economy improve. Based on these mechanisms and previous research, I test the following hypotheses:

*H3a*: Over time, respondents who improved their personal economic situation or their perception of it on average increased in European solidarity.

*H3b*: Over time, respondents who improved their perception of their country's economic situation on average increased in European solidarity.

Recent research shows that regional exposure to crises can affect European solidarity. Residents in crisis-hit regions may see themselves as potential beneficiaries of European solidarity, and thus increase their support for it (Hetzer and Burgoon, 2024). Even in Germany, crises may raise the perceived benefits of European solidarity, increasing its support. This mechanism most likely takes effect in economic crises, but may also operate in health crises, where basic needs take priority, and in immigration crises, where group competition is salient. Empirically, Hetzer and Burgoon (2024) find that regions hit harder by the COVID-19 pandemic showed greater fiscal solidarity, but neither the 2008 economic crisis nor the post-2015 immigration inflows had significant effects. Similarly, Reinl et al. (2024a) report higher European solidarity in the health sector in regions hit harder by COVID-19. Based on these mechanisms and previous research, I test the following hypotheses:

*H4a*: Respondents living in regions which experienced more economic hardship on average increased in European solidarity.

*H4b*: Respondents living in regions which experienced a more severe COVID-19 pandemic on average increased in European solidarity.

*H4c*: Respondents living in regions which experienced greater increases in refugee immigration on average increased in European solidarity.

## Methods and data

### PSDE and GLES datasets

This research draws on two panel datasets from Germany, the German Longitudinal Election Study (GLES) and the Political Solidarity in Germany (PSDE) dataset. Single-country data is used because no cross-national panel survey measures all relevant concepts. Germany is an influential case for studying European solidarity: it is both the largest net contributor to European solidarity funds (Bechtel et al., 2014) and has often opposed policies of European solidarity (Howarth and Schild, 2021). In 2020 however, Germany broke away from its support for austerity, and together with France it initiated an economic recovery plan that became NextGenerationEU (Waas and Rittberger, 2024). Understanding how and when Germans adjust their European solidarity offers insights into how citizens of the EU's largest member country may shape the future of Social Europe. Germany's federal structure also enables to analyse regional crises impacts, given substantial state-level heterogeneity in economic growth (Lehmann and Wikman, 2025), COVID-19 mortality (Doblhammer et al., 2021), and refugee inflows (Sauer et al., 2023).

Findings from Germany are most likely to generalise to other north-western European countries, such as France, the Netherlands, and Sweden. Recent survey experiments show that, during the COVID-19 pandemic and contrary to their reputation, Germans expressed more European solidarity than citizens of other states commonly seen as frugal (Oana and Truchlewski, 2024). Like respondents in France, the Netherlands and Sweden, however, Germans tend to direct their solidarity toward an inner circle of countries in Western Europe (Oana and Truchlewski, 2024). The way economic and cultural political orientations relate to European solidarity in Germany also closely match patterns observed in other north-western European countries, but differ from those in central and southern Europe (Visconti and Pellegata, 2025).

**Figure 2.**
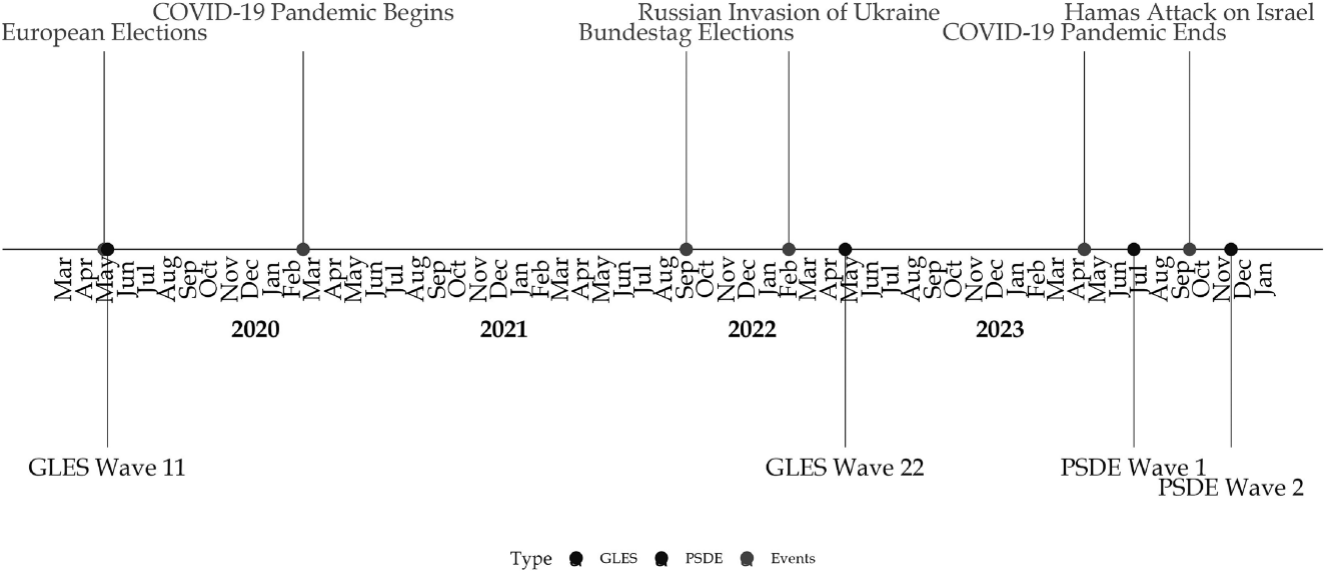
Timeline of GLES and PSDE survey waves and notable events*.*

Building on the concept of data triangulation to maximise theoretical insights (Denzin, 2009), this article relies on two panel datasets, described in detail in the Online appendix. The GLES fielded Wave 11 in May/June 2019, with 9,503 complete interviews (GLES, 2023), and Wave 22 in May 2022 with 11,786 interviews (GLES, 2022). I use data from 6,289 respondents who participated in both waves in the analysis (34 % attrition rate). Wave 11 data was collected in close proximity to the 2019 European Parliament elections, which may have increased European identification and politicised European solidarity (see Figure 2 for an overview of the study period). Between the two GLES panel waves, the COVID-19 pandemic began and eventually subsided; the Bundestag election took place, and Russia launched its full-scale invasion of Ukraine, events which affected European solidarity (Oana et al., 2025; Russo, 2023b). These were far from normal times. I therefore use the PSDE dataset to investigate stability and change in more typical conditions (Goerres, 2021).^4^ Wave 1 of this survey was completed by 2,006 respondents in July 2023, and Wave 2 by 1,519 respondents in December 2023 (24% attrition rate). Both datasets are based on non-probability samples from Germany. As the analysis aims to understand dynamics over time, and not to estimate the prevalence of attitudes in the population, using non-probability samples is unlikely to bias the analysis.

### Operationalisations

In the PSDE data, I measure European solidarity as support for welfare solidarity and European social citizenship (see Table 2 for wording). Identity is measured with two separate items that ask about individuals’ attachment to Germany and to Europe. The two value-based dimensions are operationalised differently: economic political orientation comes from two items, averaged into an index. The items ask whether the state should be responsible for reducing income inequality, and whether taxes should increase to expand social services. I measure transnational political orientation as support for facilitating immigration. The utilitarian-based explanation is tested using personal income. Control variables include left–right orientation, political trust, and general trust.

**Table 2.** Operationalisation of European solidarity dimensions in PSDE and GLES datasets**.**

Dimension	Item wording	Response scale
PSDE		
European social citizenship	In the future, immigrants from the EU should have the same entitlement to social welfare as people born in Germany.	1 Disagree 4 Agree
Welfare solidarity	I am willing to support state support from the European Union for other people in the EU, even if I do not benefit from it.	1 Don’t agree at all 5 Agree fully
GLES		
Territorial solidarity	The European Union should do more to harmonise living conditions between EU countries.	1 Strongly disagree 5 Strongly agree
Fiscal solidarity	Germany should provide financial support for EU member states experiencing great economic and financial difficulties.	1 Strongly disagree 5 Strongly agree

In the GLES data, I measure European solidarity as territorial solidarity and fiscal solidarity. The operationalisation of identity-based explanations is the same as in the PSDE data. For the value-based explanation, a third item – unavailable in the PSDE data – is added to the index of economic political orientation, which asks whether the state should be involved in the economy. Transnational political orientation is measured with three items in an index: support for immigration policy, support for further European integration, and whether foreigners in Germany should fully assimilate or be allowed to maintain their culture. For the utilitarian explanation, I use perceptions of personal economic conditions and of the national economy in Germany. Control variables include left–right orientation, general political interest and European politics interest, support for EU border protection,^5^ support for increasing Germany's defence spending, and preferences over climate protection against economic growth.

In the analysis of the GLES data, I also include regional crisis indicators at the NUTS-1 level (German states), obtained from the German Statistical Office. The PSDE dataset does not allow this analysis, as it spans only five months in 2023, with no substantive variation in crisis indicators between waves. To measure the impact of economic crisis, regional disposable household income per capita in 2019 and 2022 is used. To measure the impact of the COVID-19 pandemic crisis, I use regional excess mortality, comparing a baseline mortality from June 2016 to May 2019 in Wave 11 to the mortality from June 2019 to May 2022 (between survey waves) in Wave 22. The impact of the so-called ‘refugee crisis’ is measured as the regional stock of asylum seekers (*Schutzsuchende*) in 2019 and 2022, regardless of their legal status.

All dependent and independent variables are rescaled to range from 0 to 1. A value of 1 indicates European solidarity, strong identification, left-wing economic political orientation, a transnationalist political orientation, a positive perception of the economic situation, a right position in left–right orientation, and agreement with the control variables. The Online appendix includes the exact wording, distributions, pairwise correlations, and measures of stability and change for all variables.

### Empirical strategy

Multinomial logistic regression models allow to analyse who remains stable (i.e. shows no change in levels), increases or decreases in European solidarity over time.

Following Lundberg et al. (2021), I define the theoretical estimand for the analysis of change in European solidarity: it is the difference in person *i*'s support for European solidarity if they changed in the core independent variables versus if they did not. The target population about which this study seeks to generalise is the German adult resident population. The empirical estimand is the difference in the average change in European solidarity for those who changed in the core independent variables versus those who did not change. Two-way fixed-effects models with respondent and wave fixed effects (Baltagi, 2021: 48–49) allow to estimate this effect. These models use the panel structure of the data and control for all time-constant variance between respondents and for shared trends in European solidarity over time, thus reducing omitted-variable bias. The models use only within-individual variation in the estimation, which reduces the effective sample size, and prohibits analysing time-constant variables such as gender (Vaisey and Miles, 2017: 47). Still, after considering alternatives, these models are best suited for estimating the within-individual effect of interest.^6^

In all fixed-effects models, standard errors are clustered at the level of respondents. Results from unweighted models are reported in the main text, as weights can increase bias in panel data analysis instead of reducing it (Kristman et al., 2005). There are no substantive differences with models that use attrition weights and models without wave fixed effects. Additional models that allow effects to vary between East and West Germany (following Auspurg et al., 2019) show only minor differences. The Online appendix also contains cross-sectional models for each dimension of European solidarity in each wave.

## Stability and change in European solidarity

Table 3 reports descriptive statistics for levels of European solidarity and for its stability and change across waves. Support differs by dimension: it is lowest for European social citizenship (PSDE) and highest for territorial solidarity (GLES). Beyond the theoretical reasons for analysing each dimension of European solidarity separately, these differences in levels, and the only modest pairwise correlations between the dimensions (*r* = .36 to *r* = .51, see Online appendix) provide an empirical reason to treat them separately.

**Table 3.** Change in European solidarity in PSDE and GLES datasets**.**

European solidarity dimension	Mean level first wave	Mean level second wave	Mean change	Mean absolute change	% decrease	% stability	% increase
PSDE							
European social citizenship	0.37	0.31	−0.06	0.18	29.1%	56.5%	14.4%
Welfare solidarity	0.60	0.56	−0.04	0.20	33.2%	44.2%	22.6%
GLES							
Territorial solidarity	0.62	0.59	−0.03	0.17	31.3%	47.4%	21.4%
Fiscal solidarity	0.41	0.43	0.01	0.15	22.6%	51.6%	25.7%

*Note:* Intra-individual levels and change in European solidarity in PSDE waves from July 2023 to December 2023, and GLES waves from May/June 2019 to May 2022. Mean levels and mean absolute change normalised to the range [0 to +1], mean change normalised to the range [-1 to +1].

Across dimensions, 44–57% of respondents remain stable, i.e. they choose the same response category in both waves (see the ‘% Stability’ column in Table 3). A substantial share of respondents changes their views: except for fiscal solidarity, more respondents decrease than increase their support (see the ‘% Decrease’ and ‘% Increase’ columns in Table 3). While mean changes are small, paired t-tests show significant within-individual change between waves in all dimensions of European solidarity (see Online appendix). In absolute terms, respondents changed on average by about 15% to 20% of the scale range, equating to about one scale point. This within-individual variance allows the later use of fixed-effects models to analyse change.

**Figure 3.**
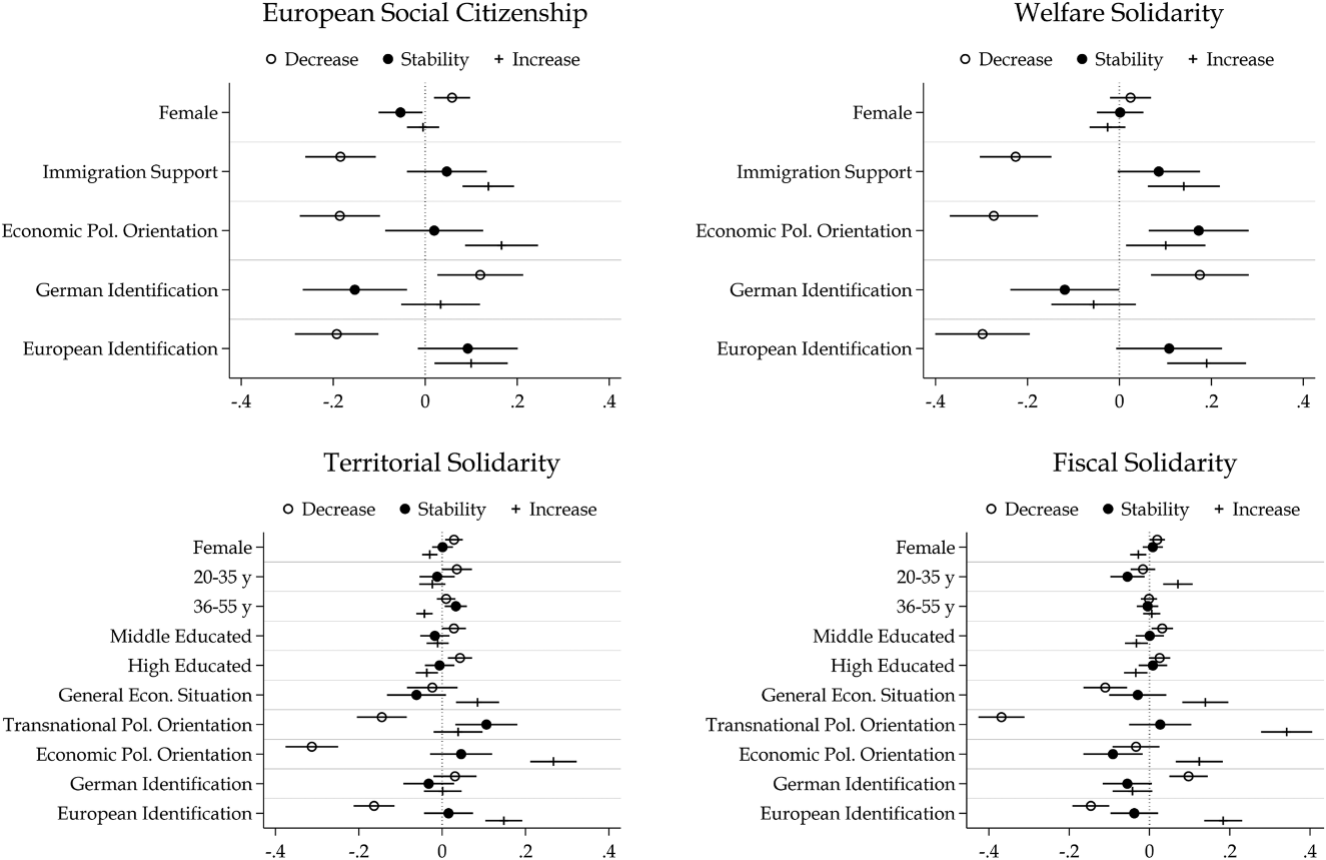
Average marginal effects (AMEs) on the probability of decrease/stability/increase in European solidarity.

*Note:* Top row for PSDE data and bottom row for GLES data. Reference categories are male, older than 56 years, and lower-educated. Reading example: female respondents are predicted to have a six percentage points higher probability of a decrease in support for European social citizenship compared to male respondents.

Figure 3 shows average marginal effects from multinomial logistic regression models that predict whether respondents decrease, stay stable, or increase their European solidarity. The Online appendix includes the full multinomial logistic regression tables. In the PSDE data, likelihood ratio tests indicate that age, education, personal income, and political and social trust – separately and jointly – do not significantly improve the model fit and are therefore not included in the displayed models. In the GLES data, personal economic conditions, and interest in general politics as well as in European politics – separately and jointly – do not significantly improve the model fit, and are therefore not included in the displayed models.

Taken together, sociodemographic characteristics do not substantially predict whether respondents remain stable or change their level of European solidarity. In three of the four dimensions, women and men show similar stability. However, in all models except for welfare solidarity, women are significantly more likely to reduce their solidarity. Compared to respondents aged 56 and older, middle-aged respondents are more likely to maintain their level of territorial solidarity, while young respondents are less likely to maintain their level of fiscal solidarity. In the GLES data, higher-educated respondents are just as likely as lower-educated respondents to maintain their level of solidarity, but they are slightly more likely to lower their solidarity. Respondents who perceive the economic situation in Germany more positively in the first GLES wave are more likely to increase in both territorial and fiscal solidarity and less likely to decrease in fiscal solidarity.

Initial levels of transnational and economic political orientation strongly predict who stays stable or changes. In all models, respondents with a more transnationalist political orientation – measured in the PSDE data as support for immigration – are significantly less likely to decrease in European solidarity. They more often maintain their level of welfare and territorial solidarity and are more likely to increase in all dimensions, except for territorial solidarity. The estimated average marginal effects are substantial: for example, respondents with the most transnationalist orientation are, on average, about 38 percentage points less likely to decrease their fiscal solidarity than those with the least transnationalist orientation. Economic political orientation also matters. In all dimensions except for fiscal solidarity, economically left-leaning respondents are less likely to decrease and more likely to increase their solidarity. They are also more likely to maintain their level of welfare solidarity, but less likely to maintain their fiscal solidarity.

Identifying with Germany or Europe can both promote and inhibit changes in European solidarity. Respondents who identify more strongly with Germany in the first wave are more likely to decrease their solidarity, except for territorial solidarity, and they tend to be less stable in all dimensions. In contrast, stronger identification with Europe is positively associated with a lower risk of decreasing and a higher chance of increasing in every dimension of European solidarity. Except for fiscal solidarity, identifying with Europe also tends to be associated with a more stable attitude.

**Figure 4.**
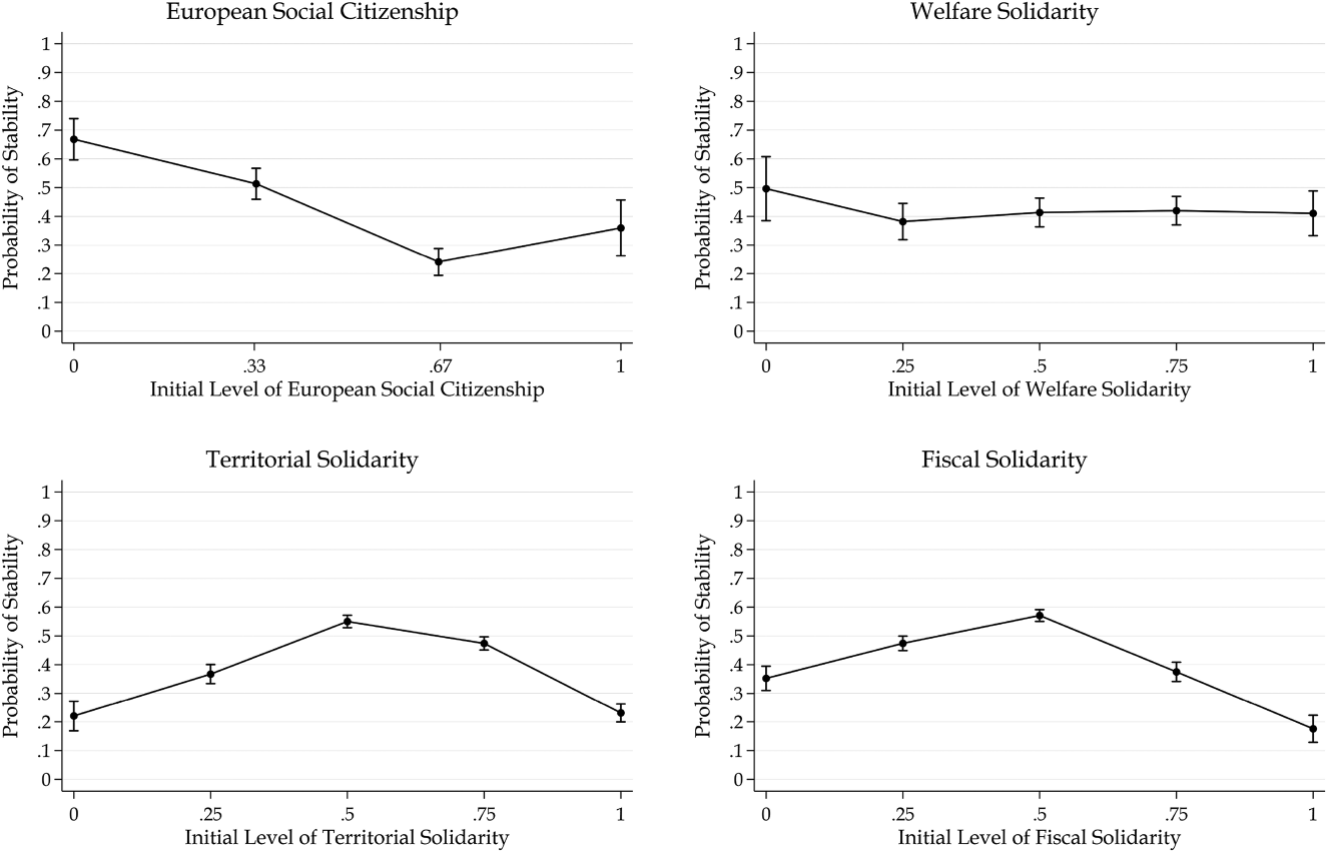
Predicted probabilities of stability in European solidarity against initial levels of European solidarity.

*Note:* Top row for PSDE data and bottom row for GLES data. Reading example: respondents with an initial minimal level of European social citizenship support have a predicted probability of stability in European social citizenship support of 68%.

All models include quadratic terms to test whether attitudes at the extremes of European solidarity and political orientation are more stable. Figure 4 plots the predicted probability of remaining stable in each dimension of European solidarity as a function of its initial level. For European social citizenship, respondents with low initial support remain more stable, and stability does not increase at the extremes. For welfare solidarity, stability is consistent across its initial levels. In the GLES data, the pattern runs counter to expectations: territorial and fiscal solidarity are less stable at extreme levels. Respondents in the middle category have a probability of stability of more than 50%, whereas at the extremes, the probability of stability drops to about 20%. Similarly, stability does not increase at extreme values of economic and transnational political orientation (see the Online appendix).

In summary, stability in European solidarity appears across sociodemographic groups. Sociodemographic factors such as gender, age, education, and income mostly do not improve model fit and show only small effects. What matters are respondents’ initial political orientations, identities, and solidarity levels. Individuals with a more transnationalist political orientation are less likely to reduce their European solidarity, more likely to increase it, and overall show more stability. The same pattern holds for those on the economic left. Respondents who strongly identify with Germany are more likely to decrease their European solidarity and show less stability. A stronger identification with Europe is associated with a lower risk of a decrease and a higher probability of an increase in European solidarity. Finally, respondents at the extremes of European solidarity and political orientation are not more likely to maintain their level of European solidarity.

## Changes in European solidarity over time

Next, I investigate the factors that explain intra-individual change in European solidarity over time. Table 4 shows the results from two-way fixed-effects regression models using the PSDE data for support for European social citizenship (model 1 and 2) and European welfare solidarity (model 3 and 4). Model 1 and 2, as well as model 3 and 4, differ in the inclusion of control variables. Table 4 also shows the results for territorial and fiscal solidarity using the GLES data.^7^ With 3% to 7% of the variance within individuals explained, the models explain only a small part of the variation over time. However, this is not untypical of individual fixed-effects regression models (see e.g. Bobzien and Kalleitner, 2021).

**Table 4.** Two-way fixed-effects regression models with PSDE and GLES data. See full tables in the Online appendix.

	Eur. social citizenship		Welfare solidarity	
Model	(1)	(2)	(3)	(4)
Identity-based explanation				
German identification	0.04 (0.04)	0.04 (0.04)	0.10* (0.05)	0.11* (0.05)
European identification	−0.00 (0.05)	−0.01 (0.05)	0.08 (0.04)	0.07 (0.04)
Value-based explanation				
Economic orientation	−0.03 (0.04)	−0.04 (0.04)	0.07 (0.05)	0.06 (0.05)
Immigration support	0.11** (0.04)	0.10** (0.04)	0.08* (0.04)	0.07 (0.04)
Utilitarian explanation				
Personal income	0.04 (0.09)	0.06 (0.09)	−0.10 (0.12)	−0.08 (0.12)
Constant	0.31*** (0.05)	0.30*** (0.07)	0.44*** (0.06)	0.45*** (0.08)
Control variables included?	No	Yes	No	Yes
Respondents	1455	1455	1455	1455
*R*^2^ within	0.06	0.07	0.03	0.04

	Territorial solidarity		Fiscal solidarity	
Model	(5)	(6)	(7)	(8)
Identity-based explanation				
German identification	−0.03 (0.02)	−0.04* (0.02)	−0.02 (0.02)	−0.02 (0.02)
European identification	0.07*** (0.02)	0.06** (0.02)	0.09*** (0.02)	0.08*** (0.02)
Value-based explanation				
Economic orientation	0.14*** (0.03)	0.12*** (0.03)	0.12*** (0.02)	0.12*** (0.02)
Transnational orientation	0.17*** (0.03)	0.15*** (0.03)	0.25*** (0.03)	0.23*** (0.03)
Utilitarian explanation				
Personal economic situation	0.01 (0.02)	0.01 (0.02)	0.02 (0.02)	0.01 (0.02)
General economic situation	0.03 (0.02)	0.02 (0.02)	0.06** (0.02)	0.06*** (0.02)
Regional household income	−0.01 (0.01)	−0.01 (0.01)	0.01 (0.01)	0.01 (0.01)
Regional mortality	−0.06* (0.03)	−0.05 (0.03)	−0.01 (0.03)	−0.00 (0.03)
Regional refugee inflow	0.04 (0.03)	0.04 (0.03)	−0.03 (0.02)	−0.03 (0.02)
Constant	0.43*** (0.03)	0.37*** (0.04)	0.15*** (0.03)	0.16*** (0.03)
Control variables included?	No	Yes	No	Yes
Respondents	8865	8865	8873	8873
R^2^ within	0.04	0.06	0.05	0.07

* *p* < .05, ** *p* < .01, *** *p* < .001.

The evidence is mixed on how shifts in identifying with Germany or with Europe affect European solidarity (Hypothesis 1). Contrary to expectations, the PSDE data show that respondents who increasingly identify with Germany tend to become more solidaric. This increase is statistically significant for welfare solidarity. However, the effect moves in the opposite direction in the GLES data and is significant for territorial solidarity only when control variables are included. The evidence is more in line with expectations for identification with Europe: respondents who grow more attached to Europe tend to increase their welfare, territorial, and fiscal solidarity. The effect is not statistically significant for welfare solidarity, and is close to zero for European social citizenship.

Value-based factors, economic and transnational political orientations, drive most intra-individual change in European solidarity (Hypothesis 2). Transnational political orientation shows substantively stronger and more consistent effects than economic political orientation. Moving left economically is associated with greater territorial and fiscal solidarity, but the effect is not statistically significant for welfare solidarity, and is substantively zero for European social citizenship. In contrast, becoming more transnationalist – measured in the PSDE data as support for immigration – is significantly associated with greater European solidarity for all dimensions except for welfare solidarity in model 4. The effect is substantial: for example, respondents who increased in transnational political orientation by 10 percentage points (about the average absolute change) are predicted to rise by about 2.3 percentage points in fiscal solidarity.^8^

Material personal and sociotropic self-interest, as well as the regional crisis impacts, do not substantially explain changes in European solidarity (Hypotheses 3 and 4). Neither personal income (PSDE) nor the perception of the personal economic situation (GLES) are significantly associated with European solidarity. Respondents who become more optimistic in their perception of the national economy tend to become more solidaric in the GLES data, although this effect is only statistically significant for fiscal solidarity. Regional household income and regional refugee inflows show no significant associations. An alternative specification using regional GDP per capita to operationalise economic conditions yields only a weak significant effect for territorial solidarity (see the Online appendix). Higher increases in regional mortality are associated with lower territorial solidarity in model 5, but the effect is no longer statistically significant after control variables are included.

Among the control variables, respondents in both datasets who move to the right on the left–right scale show lower support for European solidarity. In the PSDE data, increases in trust are associated with greater European solidarity. In the GLES data, other control variables show inconsistent effects between territorial solidarity and fiscal solidarity (see the full regression tables in the Online appendix).

## Conclusion and discussion

Using two German panel datasets, this article examined identity-based, value-based, and utilitarian explanations for intra-individual stability and change in European solidarity over time. A large share of respondents – 44%–57% over 5 months in 2023 (PSDE) and 47%–52% over 3 years from 2019 to 2022 (GLES) – maintained stable levels of European solidarity. This stability persisted across sociodemographic groups despite major events such as economic downturn, the COVID-19 pandemic and Russia's full-scale invasion of Ukraine. For many Germans, European solidarity appears relatively hard-wired. Still, declines in solidarity were more likely among those who identified strongly with Germany but identified weakly with Europe, and among those with right-leaning economic and transnational political orientations.

This study provides the first test of identity-based, value-based, and utilitarian explanations for intra-individual change in European solidarity. The analysis highlights that value-based factors, especially transnational political orientation, are the main driver of changes in European solidarity. When individuals become more transnationalist, their support for European solidarity rises across all dimensions under study. Economic orientation matters too, but only significantly for territorial and fiscal solidarity. Together, these results underscore how strongly the transnational cleavage shapes European solidarity (Hooghe and Marks, 2018; Kriesi et al., 2024).

There is mixed evidence for the identity-based explanation: stronger European identification was associated with increased territorial and fiscal solidarity, and tentatively associated with welfare solidarity. National identification, however, showed contradictory effects, positively associated with welfare solidarity but negatively with territorial solidarity. Put differently: respondents who came to feel more German became more willing to support European citizens in need, but less willing to reduce wealth inequalities between European countries. These findings point to gaps in current theory relating to divergent effects across the dimensions of European solidarity, and highlight the importance of analysing distinct solidarity dimensions separately.

These results also add nuance to recent claims that utilitarian factors dominate in explaining levels of European solidarity (Russo, 2023a: 571). Personal and sociotropic material self-interest, and the regional impacts of economic crisis, COVID-19, and refugee inflows had little effect on intra-individual change. However, the operationalisation of utilitarian factors remained limited in this study, and I could not evaluate additional considerations, such as deservingness criteria (Heermann et al., 2023; Van Oorschot, 2000).

Methodologically, this study improves on previous cross-sectional research by using fixed-effects regression models on panel data. This reduces time-invariant omitted-variable bias, and allows for cautious causal interpretations of the results. Differences between the PSDE and GLES datasets highlight the benefits of combining multiple datasets: the GLES with its longer timeframe and larger sample captures more substantive variation and achieves higher precision, while the shorter PSDE panel shows that shifts in transnational political orientation already matter over a span of months. The PSDE data also provides insights into European social citizenship and welfare solidarity, dimensions of European solidarity not covered by the GLES.

Despite these advances, the study has several limitations. First, it covers only four dimensions of European solidarity, omitting others such as refugee solidarity (Gerhards et al., 2019) and preferences on the decision-making level for social policy (Baute et al., 2018). Second, the analysis is limited to Germany, an influential but specific context. The findings are most likely to generalise to north-western European countries rather than to southern, central and eastern member states. Third, while fixed-effects models remove time-invariant confounding, time-varying confounders and measurement error may still introduce bias. Fourth, with only two waves per dataset, reverse causality could not be fully assessed, though cross-lagged panel models support the theorised direction (see the Online appendix). Fifth, attitudes can be shaped by political leaders and parties, but the short panels and relative stability of party positions during the study period preclude analysing such effects. Future research should use cross-national, multi-wave panel surveys with a comprehensive set of variables collected over longer timeframes. This would extend our understanding of the dynamics of solidarity.

This article shows that value-based explanations, especially transnational political orientation, are central for understanding why European solidarity stays stable for some individuals and shifts for others. Its findings also provide insights for policymakers: Sustaining solidarity may require to reach those most prone to decline: i.e. individuals with strong national identities but weak European identities, and those with right-leaning economic and transnational orientations.

## Supplemental Material

sj-docx-1-eup-10.1177_14651165261423107 - Supplemental material for What drives European solidarity? Evidence on identity-based, value-based, and utilitarian explanations Supplemental material, sj-docx-1-eup-10.1177_14651165261423107 for What drives European solidarity? Evidence on identity-based, value-based, and utilitarian explanations by Jakob J Eicheler in European Union Politics

sj-zip-2-eup-10.1177_14651165261423107 - Supplemental material for What drives European solidarity? Evidence on identity-based, value-based, and utilitarian explanations Supplemental material, sj-zip-2-eup-10.1177_14651165261423107 for What drives European solidarity? Evidence on identity-based, value-based, and utilitarian explanations by Jakob J Eicheler in European Union Politics

sj-zip-3-eup-10.1177_14651165261423107 - Supplemental material for What drives European solidarity? Evidence on identity-based, value-based, and utilitarian explanations Supplemental material, sj-zip-3-eup-10.1177_14651165261423107 for What drives European solidarity? Evidence on identity-based, value-based, and utilitarian explanations by Jakob J Eicheler in European Union Politics

## Notes

1. With the exception of two studies, which both focus on specific conditions of the COVID-19 pandemic and which only consider a restricted set of explanations: Bobzien & Kalleitner (2021) study Austria, and as independent variables, they only consider economic interest, COVID-19 threat perceptions, and preferences for a more central organisation at the EU level. Goldberg et al. (2021) only consider media usage, trust in institutions (including the EU), and the economic prospects in the Netherlands.

2. Due to data constraints in both the PSDE and GLES data, I am unable to examine further dimensions, such as refugee solidarity, another dimension of European solidarity in the framework by Gerhards et al. (2019).

3. Other factors shown to affect European solidarity include basic values (Reinl et al., 2024b), EU membership attitudes and trust in the EU (Russo, 2023a), transnational experiences (Ciornei and Recchi, 2017), center–periphery divides (Clasen, 2024), and domestic institutional quality (Bauhr and Charron, 2018).

4. The Ethics Review Board of Human-Centered Computing and Cognitive Science at the University of Duisburg-Essen pre-approved the research design (No 2106PWGA4168).

5. While related to transnationalism, this item taps two preferences – support for EU-level policy-making (transnationalist) and support for stronger border controls (nationalist) – and is therefore used as a control variable.

6. See the Online appendix for a discussion of and the results from alternative models. These include cross-sectional and weighted models, fixed effects models including only individual fixed effects, and models differentiating between East and West Germany.

7. See the Online appendix for one-way fixed effects models that do not include the wave fixed effects. These show results almost identical to the two-way fixed effects models reported in the main text.

8. As all variables are rescaled to the 0–1 range, this typical effect can be calculated as the mean absolute change in transnational orientation multiplied by its estimated coefficient in model 8: 0.1 × 0.23 = 0.023, corresponding to a 2.3 percentage point increase in fiscal solidarity.
