# Supplementary material for: What drives European solidarity? Evidence on identity-based, value-based, and utilitarian explanations
Source: Eur Union Polit. 2026 Mar 12;27(2):325–48. doi: 10.1177/14651165261423107 (PMC13218604; doi:10.1177/14651165261423107)
Supplement: sj-docx-1-eup-10.1177_14651165261423107 - Supplemental material for What drives European solidarity? Evidence on identity-based, value-based, and utilitarian explanations [file sj-docx-1-eup-10.1177_14651165261423107.docx]

Online appendix

What Drives European Solidarity?

Evidence on Identity-Based, Value-Based, and
Utilitarian Explanations

Jakob Jonathan Eicheler

# Contents

[Contents 1](#_Toc215433774)

[A.1 Details on the PSDE and GLES datasets 3](#_Toc215433775)

[A.2 Information on variables included in regressions 4](#_Toc215433776)

[A.2.1 PSDE 4](#_Toc215433777)

[A.2.1.1 Operationalisation of variables 4](#_Toc215433778)

[A.2.1.2 Sample Statistics in PSDE 6](#_Toc215433779)

[A.2.1.3 Table of bivariate correlations in PSDE 6](#_Toc215433780)

[A.2.1.4 Table of intra-individual stability and change in PSDE 7](#_Toc215433781)

[A.2.2 GLES 8](#_Toc215433782)

[A.2.2.1 Operationalisation of variables 8](#_Toc215433783)

[A.2.2.2 Regional Crisis Impact in GLES 10](#_Toc215433784)

[A.2.2.3 Sample Statistics in GLES 11](#_Toc215433785)

[A.2.2.4 Table of bivariate correlations in GLES 12](#_Toc215433786)

[A.2.2.5 Table of intra-individual stability and change in GLES 13](#_Toc215433787)

[A.3 Discussion of alternative modelling approaches 14](#_Toc215433788)

[A.4 Cross-Sectional and Two-Way Fixed Effects Models 16](#_Toc215433789)

[A.4.1 PSDE 16](#_Toc215433790)

[A.4.1.1 Cross-Sectional Model for European Social Citizenship 16](#_Toc215433791)

[A.4.1.2 Cross-Sectional Model for Welfare Solidarity 17](#_Toc215433792)

[A.4.1.3 Two-Way Fixed-Effects Regression Models with PSDE Data 18](#_Toc215433793)

[A.4.2 GLES data 19](#_Toc215433794)

[A.4.2.1 Cross-Sectional Model for Territorial European Solidarity 19](#_Toc215433795)

[A.4.2.2 Cross-Sectional Model for Fiscal Solidarity 20](#_Toc215433796)

[A.4.2.3 Two-Way Fixed-Effects Regression Models with GLES Data 21](#_Toc215433797)

[A.5 Robustness Checks 22](#_Toc215433798)

[A.5.1 PSDE 22](#_Toc215433799)

[A.5.1.1 Models without wave fixed effects (one-way fixed effects) 22](#_Toc215433800)

[A.5.1.2 Models with weights applied 23](#_Toc215433801)

[A.5.1.3 Models with east/west Germany interactions 23](#_Toc215433802)

[A.5.2 GLES data 25](#_Toc215433803)

[A.5.2.1 Models without wave fixed effects (one-way fixed effects) 25](#_Toc215433804)

[A.5.2.2 Models with weights applied 27](#_Toc215433805)

[A.5.2.3 Models with east/west Germany interactions 29](#_Toc215433806)

[A.5.2.3 Models with GDP per capita instead of disposable income 30](#_Toc215433807)

[A.5.2.4 Models with Alternative Operationalisations of Transnational Orientation 32](#_Toc215433808)

[A.6 Paired t-Tests of Change in European Solidarity 34](#_Toc215433809)

[A.6.1 PSDE 34](#_Toc215433810)

[A.6.2 GLES 34](#_Toc215433811)

[A.7 Multinomial Logistic Regression 35](#_Toc215433812)

[A.7.1 Stability Across Initial Levels of Political Orientation 35](#_Toc215433813)

[A.7.2 PSDE 37](#_Toc215433814)

[A.7.3 GLES 39](#_Toc215433815)

[A.8 Cross-lagged panel models 41](#_Toc215433816)

[A.8.1 PSDE 41](#_Toc215433817)

[A.8.2 GLES 45](#_Toc215433818)

[References 50](#_Toc215433819)

# A.1 Details on the PSDE and GLES datasets

I use data from an original panel survey conducted as part of the POLITSOLID project, the Political Solidarity in Germany panel survey (PSDE). The first wave of the survey was completed by 2,006 respondents in July 2023, and 1,519 respondents completed the second wave in December 2023 (24 % attrition). The respondents were provided by the forsa.omninet panel, which recruits panellists through random phone calls. There were quotas for age, gender and education, but the sample can be considered to result from a random selection of the population of Germany using the internet.

In my second analysis, I use data from the panel of the German Longitudinal Election Study (GLES, 2022, 2023), collected in the computer-assisted web interview (CAWI) mode. The GLES panel study was conducted as part of the national election study related to the 2017 federal election in Germany. Most respondents for this study were recruited via the online panel provider respondi (less than 5% via the panel provider GapFish), and are included based on a non-probability selection with quota sampling. I use panel waves 11 and 22 to conduct my analysis because these are the only waves which contain the variables to answer the research question. The data for wave 11 was collected in May/June 2019, and 9,503 complete interviews were realised. The data for wave 22 were collected in May 2022, and 11,786 complete interviews were realised. As many participants of wave 22 did not participate in wave 11, I use data from 6,289 participants.

# A.2 Information on variables included in regressions

## A.2.1 PSDE

### A.2.1.1 Operationalisation of variables

Table 1 shows the operationalisation of all variables included in the regressions (including control variables) using the PSDE dataset. Some items were reversed (see Table 1). Where concepts were measured with more than one variable, an index was created. The variables included in an index were first standardized. Then, the mean of the items for which data was available was calculated as the index. The index was calculated as long as at least one variable was non-missing.

Table 1: PSDE Operationalisation of independent, dependent and control variables

| **Concept** | **Wording** | **Scale** |
| --- | --- | --- |
| European Social Citizenship | In the future, immigrants from the EU should have the same entitlement to social welfare as people born in Germany. | 1 Disagree 4 Agree |
| Welfare Solidarity | I am willing to support state support from the European Union for other people in the EU, even if I do not benefit from it. | 1 Don’t agree at all  5 Agree fully |
| German /European Identification | How strongly do you feel attached to…?  Germany?  Europe? | 1 Strongly Attached  …  4 Not attached at all  [Reversed] |
| Economic Political Orientation | Please indicate to what extent the following issue should be the responsibility of the state.  The state should reduce the income gap between rich and poor. | 1 Not be responsible  …  5 Be responsible |
|  | Now, let us shift attention to several political issues.  Some people prefer lower taxes, although this results in less social services. Others prefer more social services, although this results in raising taxes.  What is your opinion on this issue? | 1 lower taxes, although this results in less social services  …  11 more social services, although this results in raising taxes |
| Transnational Political Orientation/ Support for Immigration | Let’s turn to the issue of immigration.  Should it be easier or more difficult for foreigners to immigrate? What is your opinion on immigration of foreigners? | 1 immigration for foreigners should be easier  …  11 immigration for foreigners should be more difficult  [Reversed] |
| Personal Income | What is your own monthly net income?  Please enter the amount in euros (e.g. € 1230.00).  Please enter an amount: | [Open Answer Field] |
| Control Variable: Left-Right Orientation | In politics people often talk of "left" and "right".  Where would you place yourself? | 1 left  …  11 right |
| Control Variable: Political Trust | For each public institution or group of people, please tell us how much you personally trust each of them. How about ...  ... the Bundestag?  ... the politicians?  ... the political parties? | 1 Do not trust at all  …  11 Trust completely |
| Control Variable: General Trust | Generally speaking: Do you believe that most people can be trusted, or that you can't be careful enough when dealing with other people? | 1 You can't be too careful  …  4 Most people can be trusted |
|  | Do you think most people try to take advantage of you when they have the opportunity, or do most people try to behave fairly? | 1 Most people try to take advantage of me  …  4 Most people try to behave fairly |
|  | And do you think that people mostly try to be helpful, or that people are mostly looking out for their own advantage? | 1 People are mostly concerned with their own advantage  …  4 People mostly try to be helpful |

### A.2.1.2 Sample Statistics in PSDE

Table 2: Summary statistics: N mean SD p25 p50 p75 in PSDE wave 1

| **Variables** | **N** | **Mean** | **SD** | **p25** | **Median** | **p75** |
| --- | --- | --- | --- | --- | --- | --- |
| European Social Citizenship | 2002 | 0.37 | 0.35 | 0 | 0.33 | 0.67 |
| Welfare Solidarity | 1996 | 0.60 | 0.32 | 0.5 | 0.75 | 0.75 |
| German Identification | 1958 | 0.74 | 0.24 | 0.67 | 0.67 | 1 |
| European Identification | 1923 | 0.64 | 0.28 | 0.67 | 0.67 | 0.67 |
| Economic Pol. Orientation | 2006 | 0.51 | 0.25 | 0.33 | 0.52 | 0.68 |
| Immigration Support | 2003 | 0.44 | 0.34 | 0.1 | 0.5 | 0.7 |
| Personal Income | 1883 | 0.26 | 0.13 | 0.17 | 0.23 | 0.33 |
| Left-Right Orientation | 1988 | 0.43 | 0.19 | 0.3 | 0.5 | 0.5 |
| Political Trust | 2006 | 0.39 | 0.25 | 0.17 | 0.4 | 0.6 |
| General Trust | 2005 | 0.51 | 0.24 | 0.33 | 0.56 | 0.67 |

Table 3: Summary statistics: N mean SD p25 p50 p75) in PSDE wave 2

| **Variables** | **N** | **Mean** | **SD** | **p25** | **Median** | **p75** |
| --- | --- | --- | --- | --- | --- | --- |
| European Social Citizenship | 1518 | 0.31 | 0.34 | 0 | 0.33 | 0.67 |
| Welfare Solidarity | 1513 | 0.56 | 0.32 | 0.25 | 0.5 | 0.75 |
| German Identification | 1486 | 0.72 | 0.23 | 0.67 | 0.67 | 1 |
| European Identification | 1461 | 0.63 | 0.27 | 0.33 | 0.67 | 0.67 |
| Economic Pol. Orientation | 1519 | 0.51 | 0.25 | 0.33 | 0.52 | 0.68 |
| Immigration Support | 1518 | 0.38 | 0.33 | 0 | 0.4 | 0.6 |
| Personal Income | 1473 | 0.27 | 0.14 | 0.18 | 0.26 | 0.33 |
| Left-Right Orientation | 1505 | 0.44 | 0.19 | 0.3 | 0.5 | 0.5 |
| Political Trust | 1518 | 0.36 | 0.24 | 0.17 | 0.37 | 0.53 |
| General Trust | 1519 | 0.52 | 0.24 | 0.33 | 0.56 | 0.67 |

### A.2.1.3 Table of bivariate correlations in PSDE

Table 4: Table of pairwise correlations in PSDE wave 1

| **Variables** | **(1)** | **(2)** | **(3)** | **(4)** | **(5)** | **(6)** | **(7)** | **(8)** | **(9)** | **(10)** |
| --- | --- | --- | --- | --- | --- | --- | --- | --- | --- | --- |
| (1) European Social Citizenship | 1.00 |  |  |  |  |  |  |  |  |  |
| (2) Welfare Solidarity | 0.47 | 1.00 |  |  |  |  |  |  |  |  |
| (3) German Identification | 0.00 | 0.10 | 1.00 |  |  |  |  |  |  |  |
| (4) European Identification | 0.33 | 0.45 | 0.38 | 1.00 |  |  |  |  |  |  |
| (5) Economic Pol. Orientation | 0.39 | 0.35 | -0.07 | 0.20 | 1.00 |  |  |  |  |  |
| (6) Immigration Support | 0.57 | 0.49 | 0.03 | 0.40 | 0.34 | 1.00 |  |  |  |  |
| (7) Personal Income | 0.01 | 0.03 | 0.06 | 0.08 | -0.12 | 0.05 | 1.00 |  |  |  |
| (8) Left-Right Orientation | -0.43 | -0.40 | 0.09 | -0.27 | -0.45 | -0.44 | 0.09 | 1.00 |  |  |
| (9) Political Trust | 0.44 | 0.41 | 0.26 | 0.46 | 0.21 | 0.48 | 0.11 | -0.28 | 1.00 |  |
| (10) General Trust | 0.35 | 0.33 | 0.15 | 0.31 | 0.21 | 0.36 | 0.09 | -0.24 | 0.41 | 1.00 |

Table 5: Table of pairwise correlations in PSDE wave 2

| **Variables** | **(1)** | **(2)** | **(3)** | **(4)** | **(5)** | **(6)** | **(7)** | **(8)** | **(9)** | **(10)** |
| --- | --- | --- | --- | --- | --- | --- | --- | --- | --- | --- |
| (1) European Social Citizenship | 1.00 |  |  |  |  |  |  |  |  |  |
| (2) Welfare Solidarity | 0.51 | 1.00 |  |  |  |  |  |  |  |  |
| (3) German Identification | 0.02 | 0.07 | 1.00 |  |  |  |  |  |  |  |
| (4) European Identification | 0.36 | 0.42 | 0.38 | 1.00 |  |  |  |  |  |  |
| (5) Economic Pol. Orientation | 0.41 | 0.39 | -0.06 | 0.22 | 1.00 |  |  |  |  |  |
| (6) Immigration Support | 0.56 | 0.49 | 0.04 | 0.37 | 0.37 | 1.00 |  |  |  |  |
| (7) Personal Income | 0.02 | 0.01 | 0.08 | 0.09 | -0.15 | 0.07 | 1.00 |  |  |  |
| (8) Left-Right Orientation | -0.43 | -0.44 | 0.06 | -0.27 | -0.49 | -0.47 | 0.12 | 1.00 |  |  |
| (9) Political Trust | 0.41 | 0.39 | 0.24 | 0.45 | 0.24 | 0.45 | 0.12 | -0.31 | 1.00 |  |
| (10) General Trust | 0.35 | 0.31 | 0.14 | 0.30 | 0.19 | 0.38 | 0.08 | -0.28 | 0.40 | 1.00 |

### A.2.1.4 Table of intra-individual stability and change in PSDE

Table 6: Table of intra-individual stability and change in PSDE

| **Variables** | **Mean level  first wave** | **Mean level  second wave** | **Mean change** | **Mean absolute change** | **%**  **Decrease** | **% Stability** | **%**  **Increase** |
| --- | --- | --- | --- | --- | --- | --- | --- |
| European social citizenship | 0.37 | 0.31 | -0.06 | 0.18 | 29.1% | 56.5% | 14.4% |
| Welfare solidarity | 0.60 | 0.56 | -0.04 | 0.20 | 33.2% | 44.2% | 22.6% |
| German Identification | 0.74 | 0.72 | -0.01 | 0.11 | 16.8% | 69.1% | 14.0% |
| European Identification | 0.64 | 0.63 | -0.01 | 0.12 | 18.1% | 66.6% | 15.2% |
| Economic Pol. Orientation | 0.51 | 0.51 | 0.00 | 0.14 | 42.3% | 16.5% | 41.3% |
| Immigration Support | 0.44 | 0.38 | -0.06 | 0.17 | 43.8% | 33.2% | 23.0% |
| Personal Income | 0.26 | 0.27 | 0.01 | 0.04 | 32.8% | 20.6% | 46.6% |
| Left-Right Orientation | 0.43 | 0.44 | 0.01 | 0.06 | 20.1% | 53.5% | 26.4% |
| Political Trust | 0.39 | 0.36 | -0.03 | 0.11 | 52.8% | 12.6% | 34.6% |
| General Trust | 0.51 | 0.52 | 0.01 | 0.13 | 33.8% | 30.0% | 36.2% |

## A.2.2 GLES

### A.2.2.1 Operationalisation of variables

Table 7 shows the operationalisation of all variables included in the regressions (including control variables) using the GLES dataset. Some items were reversed (see Table 7). Where concepts were measured with more than one variable, an index was created. The variables included in an index were first standardized. Then, the mean of the items for which data was available was calculated as the index. The index was calculated as long as at least one variable was non-missing.

Table 7: GLES Operationalisation of independent, dependent and control variables

| **Concept** | **Wording** | **Scale** |
| --- | --- | --- |
| Territorial Solidarity | Here you can find several statements with which some people agree, while others do not.  Please state to what extent you agree or disagree with each statement.  The European Union should do more to harmonise living conditions between EU countries. | 1 Strongly Disagree  …  5 Strongly Agree |
| Fiscal Solidarity | Germany should provide financial support for EU member states experiencing great economic and financial difficulties. | 1 Strongly Disagree  …  5 Strongly Agree |
| German /European Identification | People feel attached to Germany, Europe, their state and their community to a different degree.  How about you? How strongly do you feel attached to…?  Germany?  Europe? | 1 Not attached at all  …  5 Strongly Attached |
| Economic Political Orientation | Here you can find several statements with which some people agree, while others do not.  Please state to what extent you agree or disagree with each statement.  The state should take measures to reduce differences in income levels. | 1 Strongly Disagree  …  5 Strongly Agree |
|  | Now, let us shift attention to several political issues.  Some people prefer lower taxes, although this results in less social services. Others prefer more social services, although this results in raising taxes.  What is your opinion on this issue? | 1 lower taxes, although this results in less social services  …  7 more social services, although this results in raising taxes |
|  | Here you can find several statements with which some people agree, while others do not.  Please state to what extent you agree or disagree with each statement.  The state should stay out of the economy. | 1 Strongly Disagree  …  5 Strongly Agree  [Reversed] |
| Transnational Political Orientation | Let’s turn to the issue of immigration.  Should it be easier or more difficult for foreigners to immigrate? What is your opinion on immigration of foreigners? | 1 immigration for foreigners should be easier  …  7 immigration for foreigners should be more difficult  [Reversed] |
|  | Should the European unification be pushed further in order to establish a joint government soon or has the European unification already gone too far?  What is your opinion on European unification? | 1 European unification should be pushed further  …  7 European unification has already gone too far  [Reversed] |
|  | There are different views on how much foreigners should assimilate in Germany. Some people think that foreigners should completely assimilate to the German culture. Others think that foreigners should be able to live according to their own culture.  What is your opinion on this issue? | 1 foreigners should completely assimilate to the German culture  …  7 foreigners should be able to live according to their own culture |
| Personal Economic Situation | Now, let us shift attention to your economic situation.  How would you evaluate your own current economic situation? | 1 very good  2 good  3 neither good or bad  4 bad  5 very bad  [Reversed] |
| Sociotropic Economic Situation | How would you evaluate the current general economic situation in Germany? | 1 very good  2 good  3 neither good or bad  4 bad  5 very bad  [Reversed] |
| Control Variables | Quite generally, how interested are you in politics? | 1 very interested  2 somewhat interested  3 in between  4 not very interested  5 not at all interested  [Reversed] |
|  | And how interested are you in politics at federal, state, and European level?  European level | 1 very interested  2 somewhat interested  3 in between  4 not very interested  5 not at all interested  [Reversed] |
|  | Here you can find several statements with which some people agree, while others do not.  Please state to what extent you agree or disagree with each statement.  The European Union should do more to protect its external borders. | 1 Strongly Disagree  …  5 Strongly Agree |
|  | Here you can find several statements with which some people agree, while others do not.  Please state to what extent you agree or disagree with each statement.  Germany’s defence expenditure should be increased over the next few years. | 1 Strongly Disagree  …  5 Strongly Agree |
|  | Some say that the fight against climate change should definitely take precedence, even if it impairs economic growth.  Others say that the economic growth should definitely take precedence, even if it impairs the fight against climate change.  What is your personal view on this issue? | 1 fight against climate change should take precedence, even if it impairs economic growth  …  7 economic growth should take precedence, even if it impairs the fight against climate change  [Reversed] |
|  | In politics people often talk of "left" and "right".  Where would you place yourself? | 1 left  …  11 right |

### A.2.2.2 Regional Crisis Impact in GLES

In the analysis of the GLES data, I include measures of regional crisis impact at the NUTS-1 level of German states from the German Statistical Office. This section provides additional details on these variables.

For economic crisis impact, I use disposable household income in 2019 and 2022 at the regional level of German states (Table 82411-0010 DESTATIS 2024). I recode this variable so that it has a value of zero in wave 11 in 2019. I then calculate the value for wave 22 in 2022 by first calculating the percent change in disposable household income between 2019 and 2022:

Percent Change HH Income =
(Disposable Household Income in 2022 /
Disposable Household Income in 2019)-1

I then standardise the percentage change to the range 0-1 by calculating

Std. Percent Change HH Income =
(Percent Change HH Income – min(Percent Change HH Income)) /
(max(Percent Change HH Income) / min(Percent Change HH Income))

This results in a variable that is one in the region with the maximum change in disposable household income, and zero in the region with the lowest increase. The largest increase in disposable household income between 2019 and 2022 was about 12 % in North Rhine-Westphalia. Respondents from this region therefore have a value of 1 in wave 22. In contrast, disposable household income increased by only 6% in the city state of Bremen. Thus, respondents from Bremen have a value of about 0. Smaller values indicate more crisis impact of economic crisis.

For COVID-19 pandemic impact, I use total regional mortality from June 2016 to May 2019 as a baseline for the first wave. For the second wave, I use total regional mortality from June 2019 to May 2022. This total provides a less noisy measurement of regional mortality than using just the last month or year before each used wave of the GLES panel survey. I perform the same transformations as for disposable household income. The variable thus indicates the percent of the maximum increase in mortality the specific region experienced. Smaller values indicate less crisis impact.

For measuring the impact of refugee immigration on a region, I use the regional stock of all seeking refuge (“Schutzsuchende”), regardless of their legal status, in 2019 and 2022. I then perform the same transformations as for the other variables measuring regional crisis impact. The variable thus indicates the percent of the maximum increase in the stock of refugee seekers the specific region experienced. Smaller values indicate less crisis impact.

### A.2.2.3 Sample Statistics in GLES

Table 8: Summary statistics: N mean SD p25 p50 p75 in GLES wave 11

| **Variables** | **N** | **Mean** | **SD** | **p25** | **Median** | **p75** |
| --- | --- | --- | --- | --- | --- | --- |
| Territorial Solidarity | 9466 | 0.62 | 0.25 | 0.5 | 0.5 | 0.75 |
| Fiscal Solidarity | 9472 | 0.41 | 0.26 | 0.25 | 0.5 | 0.5 |
| German Identification | 9455 | 0.73 | 0.24 | 0.5 | 0.75 | 1 |
| European Identification | 9479 | 0.53 | 0.29 | 0.25 | 0.5 | 0.75 |
| Economic Pol. Orientation | 9497 | 0.59 | 0.18 | 0.5 | 0.58 | 0.72 |
| Transnational Pol. Orientation | 9496 | 0.41 | 0.23 | 0.22 | 0.44 | 0.56 |
| Personal Economic Situation | 9490 | 0.6 | 0.22 | 0.5 | 0.75 | 0.75 |
| Sociotropic Economic Situation | 9489 | 0.62 | 0.20 | 0.5 | 0.75 | 0.75 |
| Interest in EU elections | 9460 | 0.53 | 0.27 | 0.25 | 0.5 | 0.75 |
| Interest in general politics | 9486 | 0.62 | 0.27 | 0.5 | 0.75 | 0.75 |
| Support EU border protection | 9471 | 0.68 | 0.28 | 0.5 | 0.75 | 1 |
| Left–Right orientation | 8751 | 0.46 | 0.22 | 0.3 | 0.5 | 0.6 |
| Support defence spending | 9470 | 0.41 | 0.30 | 0.25 | 0.5 | 0.75 |
| Prioritise climate protection | 9474 | 0.64 | 0.28 | 0.5 | 0.67 | 0.83 |

Table 9: Summary statistics: N mean SD p25 p50 p75 in GLES wave 22

| **Variables** | **N** | **Mean** | **SD** | **p25** | **Median** | **p75** |
| --- | --- | --- | --- | --- | --- | --- |
| Territorial Solidarity | 6155 | 0.59 | 0.24 | 0.5 | 0.5 | 0.75 |
| Fiscal Solidarity | 6160 | 0.43 | 0.26 | 0.25 | 0.5 | 0.5 |
| German Identification | 6132 | 0.69 | 0.25 | 0.5 | 0.75 | 0.75 |
| European Identification | 6149 | 0.48 | 0.29 | .25 | 0.5 | 0.75 |
| Economic Pol. Orientation | 6178 | 0.58 | 0.18 | 0.5 | 0.58 | 0.69 |
| Transnational Pol. Orientation | 6176 | 0.42 | 0.23 | 0.22 | 0.44 | 0.56 |
| Personal Economic Situation | 6162 | 0.58 | 0.23 | 0.5 | 0.5 | 0.75 |
| Sociotropic Economic Situation | 6148 | 0.48 | 0.21 | 0.25 | 0.5 | 0.75 |
| Interest in EU elections | 6240 | 0.49 | 0.27 | 0.25 | 0.5 | 0.75 |
| Interest in general politics | 6271 | 0.62 | 0.27 | 0.5 | 0.75 | 0.75 |
| Support EU border protection | 6152 | 0.72 | 0.25 | 0.5 | 0.75 | 1 |
| Left–Right orientation | 5723 | 0.46 | 0.19 | 0.3 | 0.5 | 0.6 |
| Support defence spending | 6153 | 0.58 | 0.31 | 0.5 | 0.5 | 0.75 |
| Prioritise climate protection | 6162 | 0.57 | 0.30 | 0.33 | 0.5 | 0.83 |

### A.2.2.4 Table of bivariate correlations in GLES

Table 10: Table of pairwise correlations in GLES wave 11

| **Variables** | **(1)** | **(2)** | **(3)** | **(4)** | **(5)** | **(6)** | **(7)** | **(8)** |
| --- | --- | --- | --- | --- | --- | --- | --- | --- |
| (1) Territorial Solidarity | 1.00 |  |  |  |  |  |  |  |
| (2) Fiscal Solidarity | 0.36 | 1.00 |  |  |  |  |  |  |
| (3) German Identification | 0.03 | -0.06 | 1.00 |  |  |  |  |  |
| (4) European Identification | 0.30 | 0.44 | 0.33 | 1.00 |  |  |  |  |
| (5) Economic Pol. Orientation | 0.36 | 0.34 | -0.03 | 0.21 | 1.00 |  |  |  |
| (6) Transnational Pol. Orientation | 0.35 | 0.57 | -0.12 | 0.52 | 0.29 | 1.00 |  |  |
| (7) Personal Econ. Situation | 0.05 | 0.12 | 0.14 | 0.22 | -0.13 | 0.21 | 1.00 |  |
| (8) Sociotropic Econ. Situation | 0.17 | 0.25 | 0.14 | 0.29 | 0.09 | 0.33 | 0.39 | 1.00 |

Table 11: Table of pairwise correlations in GLES wave 22

| **Variables** | **(1)** | **(2)** | **(3)** | **(4)** | **(5)** | **(6)** | **(7)** | **(8)** |
| --- | --- | --- | --- | --- | --- | --- | --- | --- |
| (1) Territorial Solidarity | 1.00 |  |  |  |  |  |  |  |
| (2) Fiscal Solidarity | 0.40 | 1.00 |  |  |  |  |  |  |
| (3) German Identification | 0.09 | 0.10 | 1.00 |  |  |  |  |  |
| (4) European Identification | 0.33 | 0.50 | 0.43 | 1.00 |  |  |  |  |
| (5) Economic Pol. Orientation | 0.40 | 0.30 | 0.01 | 0.20 | 1.00 |  |  |  |
| (6) Transnational Pol. Orientation | 0.35 | 0.62 | 0.07 | 0.57 | 0.30 | 1.00 |  |  |
| (7) Personal Econ. Situation | 0.04 | 0.18 | 0.18 | 0.22 | -0.14 | 0.23 | 1.00 |  |
| (8) Sociotropic Econ. Situation | 0.18 | 0.35 | 0.19 | 0.35 | 0.12 | 0.39 | 0.38 | 1.00 |

### A.2.2.5 Table of intra-individual stability and change in GLES

Table 12: Table of intra-individual stability and change in GLES

| **Variable** | **Mean level  first wave** | **Mean level  second wave** | **Mean change** | **Mean absolute change** | **%**  **Decrease** | **% Stability** | **%**  **Increase** |
| --- | --- | --- | --- | --- | --- | --- | --- |
| Territorial Solidarity | 0.62 | 0.59 | -0.03 | 0.17 | 31.3% | 47.4% | 21.4% |
| Fiscal Solidarity | 0.41 | 0.43 | 0.01 | 0.15 | 22.6% | 51.6% | 25.7% |
| German Identification | 0.73 | 0.69 | -0.04 | 0.13 | 28.6% | 56.3% | 15.1% |
| European Identification | 0.53 | 0.48 | -0.06 | 0.15 | 33.2% | 50.8% | 16.0% |
| Economic Pol. Orientation | 0.59 | 0.58 | -0.01 | 0.11 | 43.8% | 16.5% | 39.7% |
| Transnational Pol. Orientation | 0.41 | 0.42 | 0.01 | 0.10 | 36.7% | 19.9% | 43.4% |
| Personal Economic Situation | 0.6 | 0.58 | -0.03 | 0.12 | 26.1% | 58.9% | 15.1% |
| Sociotropic Economic Situation | 0.62 | 0.48 | -0.15 | 0.18 | 52.0% | 41.5% | 6.5% |
| Interest in EU elections | 0.53 | 0.49 | -0.06 | 0.14 | 33.0% | 53.0% | 14.0% |
| Interest in general politics | 0.62 | 0.62 | -0.01 | 0.08 | 17.4% | 69.5% | 13.0% |
| Support EU border protection | 0.68 | 0.72 | 0.03 | 0.18 | 22.6% | 47.5% | 29.9% |
| Left–Right orientation | 0.46 | 0.46 | 0 | 0.08 | 27.6% | 45.5% | 26.9% |
| Support defence spending | 0.41 | 0.58 | 0.16 | 0.24 | 11.8% | 36.1% | 52.1% |
| Prioritise climate protection | 0.64 | 0.57 | -0.06 | 0.17 | 38.8% | 40.6% | 20.6% |

# A.3 Discussion of alternative modelling approaches

For the analysis of stability, multinomial logistic regression models were chosen. A simple logistic regression models would have compared those showing stability (i.e. no change) with those showing a decrease or an increase. As this can hide relevant variation (e.g. if respondents with a certain characteristic show lower risk to decrease, but higher risk to increase), a simple logistic regression model would be inadequate. Another alternative would have been ordered logistic regression. However, this assumes that the coefficients are constant between the ordered categories (Scott Long, 2014, p. 174). I expect distinct effects on stability, differing between the comparison of those decreasing to those stable and the comparison of those increasing to those stable. Therefore, ordered logistic regression would also be not suitable, and multinomial logistic regression is the optimal approach.

For the analysis of change, several alternative modelling approaches were considered, but these come with other costs. A first-difference modelling approach would have been equivalent to the fixed effects approach in the case of only two waves of panel data (Andreß et al., 2013, p. 189). Fixed effects models (without the fixed effect for panel waves) are reported in the appendix, but these do not control for the over-time change shared by all respondents. One alternative would have been the use of random effects models. But these come with the assumption that the observed predictors are not correlated with unobserved time-constant variables, which is unlikely to be the case in social science research (Vaisey & Miles, 2017, p. 47). Another alternative approach would have been the use of lagged dependent variable (LDV) models (Keele & Kelly, 2006). However, the coefficients estimated using LDV models are likely to be biased in short time series (Wilkins, 2018, p. 397), which is the case with only two waves of panel data.

I include two-way fixed effects models with and without control variables due to two potential data generating processes shown in DAG 1 and DAG 2. In DAG 1, adjusting for control variables is required to correctly adjust the model for estimating the true total effect of the core independent variables. Additionally, the control variables may allow a more precise estimation, as they reduce unexplained variance in the dependent variables measuring European solidarity. However, it is also possible that change in the independent variables could cause change in at least some of the control variables: For example, it is plausible that increases in European identification increase interest in European elections. This possibility is shown in DAG 2. As I am unable to decide which of these two causal models is correct, I estimate models 1, 3, 5, and 7 in line with DAG 1, and models 2, 4, 6 and 8 in line with DAG 2.


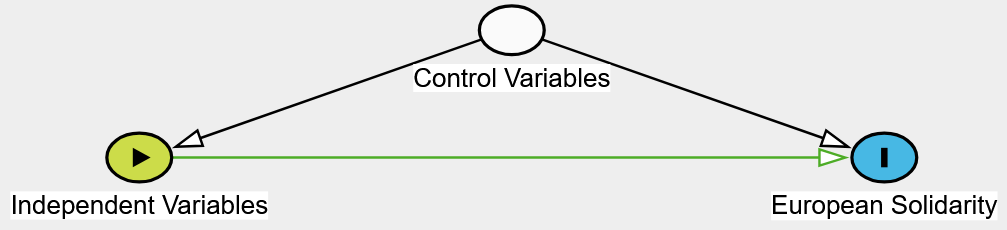


Directed Acyclic Graph 1. Adjusting for control variables makes the model correctly adjusted.


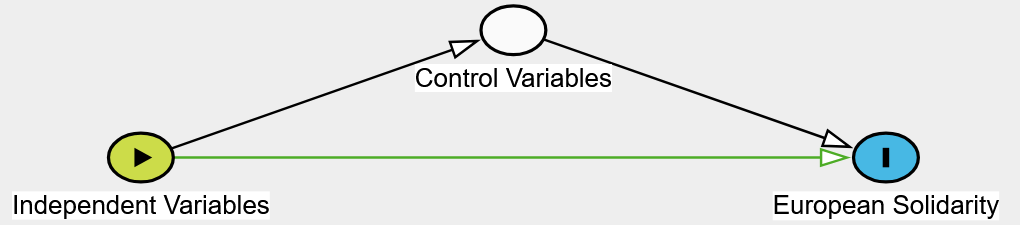
 Directed Acyclic Graph 2. Adjusting for control variables makes the model incorrectly adjusted.

# A.4 Cross-Sectional and Two-Way Fixed Effects Models

The following tables show estimates from regression models with the four dimensions of European Solidarity as the dependent variable. For each of these, cross-sectional models for each wave and two-way-fixed effects models across waves are estimated, once without and once with control variables. The cross-sectional models confirm results from previous cross-sectional research: Those identifying more with Europe, with a more left economic and a more transnationalist political orientation tend to support European Solidarity.

## A.4.1 PSDE

### A.4.1.1 Cross-Sectional Model for European Social Citizenship

Table 13: Results from cross-sectional regressions on European Social Citizenship in PSDE wave 1 and 2

|  | **(1)** | **(2)** | **(3)** | **(4)** |
| --- | --- | --- | --- | --- |
|  | **European social citizenship support**  **Wave 1** | **European social citizenship support**  **Wave 1**  **Incl. Controls** | **European social citizenship support  Wave 2** | **European social citizenship support  Wave 2**  **Incl. Controls** |
|  | b/se | b/se | b/se | b/se |
| German Identification | -0.07^*^ | -0.05 | -0.07^*^ | -0.06 |
|  | (0.03) | (0.03) | (0.03) | (0.03) |
| European Identification | 0.16^***^ | 0.14^***^ | 0.21^***^ | 0.20^***^ |
|  | (0.03) | (0.03) | (0.03) | (0.03) |
| Economic Pol. Orientation | 0.30^***^ | 0.29^***^ | 0.31^***^ | 0.31^***^ |
|  | (0.03) | (0.03) | (0.03) | (0.03) |
| Immigration support | 0.47^***^ | 0.45^***^ | 0.43^***^ | 0.40^***^ |
|  | (0.02) | (0.02) | (0.02) | (0.03) |
| Personal Income | 0.03 | -0.03 | 0.03 | -0.03 |
|  | (0.05) | (0.05) | (0.05) | (0.06) |
| Male & Diverse |  | 0.00 |  | 0.00 |
|  |  | (.) |  | (.) |
| Female |  | -0.01 |  | -0.02 |
|  |  | (0.01) |  | (0.01) |
| 18-35 years old |  | 0.02 |  | -0.00 |
|  |  | (0.02) |  | (0.02) |
| 36-55 years old |  | -0.03 |  | -0.01 |
|  |  | (0.02) |  | (0.02) |
| 56+ years old |  | 0.00 |  | 0.00 |
|  |  | (.) |  | (.) |
| 1 Low Educated |  | 0.00 |  | 0.00 |
|  |  | (.) |  | (.) |
| 2 Medium Educated |  | 0.05^**^ |  | 0.02 |
|  |  | (0.02) |  | (0.02) |
| 3 High Educated |  | 0.08^***^ |  | 0.08^***^ |
|  |  | (0.02) |  | (0.02) |
| Constant | -0.04 | -0.04 | -0.10^**^ | -0.09^*^ |
|  | (0.03) | (0.03) | (0.03) | (0.04) |
| Observations | 1776 | 1775 | 1396 | 1396 |
| R² | 0.382 | 0.395 | 0.390 | 0.399 |

^*^ *p* < 0.05, ^**^ *p* < 0.01, ^***^ *p* < 0.001

### A.4.1.2 Cross-Sectional Model for Welfare Solidarity

Table 14: Results from cross-sectional regressions on welfare solidarity in PSDE wave 1 and 2

|  | **(1)** | **(2)** | **(3)** | **(4)** |
| --- | --- | --- | --- | --- |
|  | **Welfare solidarity**  **Wave 1** | **Welfare solidarity**  **Wave 1**  **Incl. Controls** | **Welfare solidarity**  **Wave 2** | **Welfare solidarity**  **Wave 2**  **Incl. Controls** |
|  | b/se | b/se | b/se | b/se |
| German Identification | 0.00 | -0.01 | -0.04 | -0.03 |
|  | (0.03) | (0.03) | (0.03) | (0.03) |
| European Identification | 0.35^***^ | 0.34^***^ | 0.31^***^ | 0.31^***^ |
|  | (0.03) | (0.03) | (0.03) | (0.03) |
| Economic Pol. Orientation | 0.24^***^ | 0.24^***^ | 0.27^***^ | 0.26^***^ |
|  | (0.03) | (0.03) | (0.03) | (0.03) |
| Immigration support | 0.30^***^ | 0.30^***^ | 0.30^***^ | 0.29^***^ |
|  | (0.02) | (0.02) | (0.02) | (0.02) |
| Personal income | 0.02 | -0.01 | 0.01 | -0.05 |
|  | (0.05) | (0.05) | (0.05) | (0.05) |
| Male & Diverse |  | 0.00 |  | 0.00 |
|  |  | (.) |  | (.) |
| Female |  | -0.02 |  | -0.03^*^ |
|  |  | (0.01) |  | (0.01) |
| 18-35 years old |  | -0.05^*^ |  | -0.00 |
|  |  | (0.02) |  | (0.02) |
| 36-55 years old |  | -0.03 |  | 0.03 |
|  |  | (0.02) |  | (0.02) |
| 56+ years old |  | 0.00 |  | 0.00 |
|  |  | (.) |  | (.) |
| 1 Low Educated |  | 0.00 |  | 0.00 |
|  |  | (.) |  | (.) |
| 2 Medium Educated |  | 0.02 |  | 0.01 |
|  |  | (0.02) |  | (0.02) |
| 3 High Educated |  | 0.03 |  | 0.05^*^ |
|  |  | (0.02) |  | (0.02) |
| Constant | 0.12^***^ | 0.15^***^ | 0.14^***^ | 0.14^***^ |
|  | (0.03) | (0.03) | (0.03) | (0.04) |
| Observations | 1774 | 1773 | 1394 | 1394 |
| R² | 0.350 | 0.354 | 0.340 | 0.347 |

^*^ *p* < 0.05, ^**^ *p* < 0.01, ^***^ *p* < 0.001

### A.4.1.3 Two-Way Fixed-Effects Regression Models with PSDE Data

Table 15: Two-Way Fixed-Effects Regression Models with PSDE Data, Main Model

|  | **(1)** | **(2)** | **(3)** | **(4)** |
| --- | --- | --- | --- | --- |
|  | **European social citizenship support** | **European social citizenship support** | **Welfare solidarity** | **Welfare solidarity** |
|  | b/se | b/se | b/se | b/se |
| German Identification | 0.04 | 0.04 | 0.10^*^ | 0.11^*^ |
|  | (0.04) | (0.04) | (0.05) | (0.05) |
| European Identification | -0.00 | -0.01 | 0.08 | 0.07 |
|  | (0.05) | (0.05) | (0.04) | (0.04) |
| Economic Pol. Orientation | -0.03 | -0.04 | 0.07 | 0.06 |
|  | (0.04) | (0.04) | (0.05) | (0.05) |
| Immigration support | 0.11^**^ | 0.10^**^ | 0.08^*^ | 0.07 |
|  | (0.04) | (0.04) | (0.04) | (0.04) |
| Personal income | 0.04 | 0.06 | -0.10 | -0.08 |
|  | (0.09) | (0.09) | (0.12) | (0.12) |
| Wave=1 | 0.00 | 0.00 | 0.00 | 0.00 |
|  | (.) | (.) | (.) | (.) |
| Wave=2 | -0.06^***^ | -0.06^***^ | -0.03^**^ | -0.02^*^ |
|  | (0.01) | (0.01) | (0.01) | (0.01) |
| Left-Right orientation |  | -0.16 |  | -0.19^*^ |
|  |  | (0.09) |  | (0.09) |
| Political Trust |  | 0.07 |  | 0.13^*^ |
|  |  | (0.06) |  | (0.06) |
| General Trust |  | 0.11^*^ |  | 0.05 |
|  |  | (0.05) |  | (0.05) |
| Constant | 0.31^***^ | 0.30^***^ | 0.44^***^ | 0.45^***^ |
|  | (0.05) | (0.07) | (0.06) | (0.08) |
| Observations | 2736 | 2736 | 2733 | 2733 |
| Respondents | 1455 | 1455 | 1455 | 1455 |
| R² within | 0.0602 | 0.0693 | 0.0298 | 0.0396 |
| R² between | 0.255 | 0.406 | 0.332 | 0.403 |
| R² overall | 0.161 | 0.324 | 0.254 | 0.325 |

^*^ *p* < 0.05, ^**^ *p* < 0.01, ^***^ *p* < 0.001

## A.4.2 GLES data

### A.4.2.1 Cross-Sectional Model for Territorial European Solidarity

Table 16: Results from cross-sectional regressions on territorial solidarity in GLES wave 11 and 22

|  | **(1)** | **(2)** | **(3)** | **(4)** |
| --- | --- | --- | --- | --- |
|  | **Territorial Solidarity**  **Wave 11** | **Territorial Solidarity**  **Wave 11**  **Incl. Controls** | **Territorial Solidarity**  **Wave 22** | **Territorial Solidarity**  **Wave 22  Incl. Controls** |
|  | b/se | b/se | b/se | b/se |
| German Identification | 0.01 | -0.01 | 0.00 | -0.01 |
|  | (0.01) | (0.01) | (0.01) | (0.01) |
| European Identification | 0.12^***^ | 0.12^***^ | 0.14^***^ | 0.14^***^ |
|  | (0.01) | (0.01) | (0.01) | (0.01) |
| Economic Pol. Orientation | 0.38^***^ | 0.37^***^ | 0.42^***^ | 0.41^***^ |
|  | (0.01) | (0.01) | (0.02) | (0.02) |
| Transnational Pol. Orientation | 0.21^***^ | 0.21^***^ | 0.16^***^ | 0.17^***^ |
|  | (0.01) | (0.01) | (0.02) | (0.02) |
| Personal economic situation | 0.00 | 0.01 | 0.00 | 0.01 |
|  | (0.01) | (0.01) | (0.01) | (0.01) |
| Sociotropic economic situation | 0.05^***^ | 0.04^**^ | 0.03 | 0.02 |
|  | (0.01) | (0.01) | (0.01) | (0.01) |
| Male |  | 0.00 |  | 0.00 |
|  |  | (.) |  | (.) |
| Female |  | -0.04^***^ |  | -0.03^***^ |
|  |  | (0.00) |  | (0.01) |
| 20-35 years old |  | -0.04^***^ |  | -0.05^***^ |
|  |  | (0.01) |  | (0.01) |
| 36-55 years old |  | -0.04^***^ |  | -0.03^***^ |
|  |  | (0.01) |  | (0.01) |
| 56+ years old |  | 0.00 |  | 0.00 |
|  |  | (.) |  | (.) |
| 1 Low Educated |  | 0.00 |  | 0.00 |
|  |  | (.) |  | (.) |
| 2 Medium Educated |  | -0.01^*^ |  | -0.01 |
|  |  | (0.01) |  | (0.01) |
| 3 High Educated |  | -0.03^***^ |  | -0.03^***^ |
|  |  | (0.01) |  | (0.01) |
| Constant | 0.20^***^ | 0.28^***^ | 0.19^***^ | 0.26^***^ |
|  | (0.01) | (0.01) | (0.01) | (0.02) |
| Observations | 9390 | 9374 | 6071 | 6071 |
| R² | 0.214 | 0.230 | 0.241 | 0.254 |

^*^ *p* < 0.05, ^**^ *p* < 0.01, ^***^ *p* < 0.001

### A.4.2.2 Cross-Sectional Model for Fiscal Solidarity

Table 17: Results from cross-sectional regressions on fiscal solidarity in GLES wave 11 and 22

|  | **(1)** | **(2)** | **(3)** | **(4)** |
| --- | --- | --- | --- | --- |
|  | **Fiscal Solidarity Wave 11** | **Fiscal Solidarity Wave 11 Incl. Controls** | **Fiscal Solidarity Wave 22** | **Fiscal Solidarity Wave 22 Incl. Controls** |
|  | b/se | b/se | b/se | b/se |
| German Identification | -0.10^***^ | -0.09^***^ | -0.05^***^ | -0.05^***^ |
|  | (0.01) | (0.01) | (0.01) | (0.01) |
| European Identification | 0.20^***^ | 0.20^***^ | 0.19^***^ | 0.19^***^ |
|  | (0.01) | (0.01) | (0.01) | (0.01) |
| Economic Pol. Orientation | 0.26^***^ | 0.26^***^ | 0.17^***^ | 0.18^***^ |
|  | (0.01) | (0.01) | (0.01) | (0.01) |
| Transnational Pol. Orientation | 0.44^***^ | 0.43^***^ | 0.47^***^ | 0.47^***^ |
|  | (0.01) | (0.01) | (0.01) | (0.01) |
| Personal economic situation | 0.01 | 0.01 | 0.03^*^ | 0.03^*^ |
|  | (0.01) | (0.01) | (0.01) | (0.01) |
| Sociotropic economic situation | 0.08^***^ | 0.07^***^ | 0.12^***^ | 0.12^***^ |
|  | (0.01) | (0.01) | (0.01) | (0.01) |
| Male |  | 0.00 |  | 0.00 |
|  |  | (.) |  | (.) |
| Female |  | -0.01^*^ |  | -0.01^*^ |
|  |  | (0.00) |  | (0.01) |
| 20-35 years old |  | 0.03^***^ |  | 0.03^***^ |
|  |  | (0.01) |  | (0.01) |
| 36-55 years old |  | 0.01 |  | 0.02^**^ |
|  |  | (0.00) |  | (0.01) |
| 56+ years old |  | 0.00 |  | 0.00 |
|  |  | (.) |  | (.) |
| 1 Low Educated |  | 0.00 |  | 0.00 |
|  |  | (.) |  | (.) |
| 2 Medium Educated |  | -0.01 |  | -0.02^**^ |
|  |  | (0.01) |  | (0.01) |
| 3 High Educated |  | 0.01^*^ |  | -0.01 |
|  |  | (0.01) |  | (0.01) |
| Constant | -0.01 | -0.02 | -0.00 | 0.00 |
|  | (0.01) | (0.01) | (0.01) | (0.01) |
| Observations | 9397 | 9381 | 6074 | 6074 |
| R² | 0.397 | 0.400 | 0.435 | 0.437 |

^*^ *p* < 0.05, ^**^ *p* < 0.01, ^***^ *p* < 0.001

### A.4.2.3 Two-Way Fixed-Effects Regression Models with GLES Data

Table 18: Two-Way Fixed-Effects Regression Models with GLES Data, Main Model

|  | **(1)** | **(2)** | **(3)** | **(4)** |
| --- | --- | --- | --- | --- |
|  | **Territorial Solidarity** | **Territorial Solidarity** | **Fiscal Solidarity** | **Fiscal Solidarity** |
|  | b/se | b/se | b/se | b/se |
| German identification | -0.03 | -0.04^*^ | -0.02 | -0.02 |
|  | (0.02) | (0.02) | (0.02) | (0.02) |
| European identification | 0.07^***^ | 0.06^**^ | 0.09^***^ | 0.08^***^ |
|  | (0.02) | (0.02) | (0.02) | (0.02) |
| Economic Pol. orientation | 0.14^***^ | 0.12^***^ | 0.12^***^ | 0.12^***^ |
|  | (0.03) | (0.03) | (0.02) | (0.02) |
| Transnational Pol. orientation | 0.17^***^ | 0.15^***^ | 0.25^***^ | 0.23^***^ |
|  | (0.03) | (0.03) | (0.03) | (0.03) |
| Personal economic situation | 0.01 | 0.01 | 0.02 | 0.01 |
|  | (0.02) | (0.02) | (0.02) | (0.02) |
| Sociotropic economic situation | 0.03 | 0.02 | 0.06^***^ | 0.06^***^ |
|  | (0.02) | (0.02) | (0.02) | (0.02) |
| Regional household income | -0.01 | -0.01 | 0.01 | 0.01 |
|  | (0.01) | (0.01) | (0.01) | (0.01) |
| Regional mortality | -0.06^*^ | -0.05 | -0.01 | -0.00 |
|  | (0.03) | (0.03) | (0.03) | (0.03) |
| Regional refugee inflow | 0.04 | 0.04 | -0.03 | -0.03 |
|  | (0.03) | (0.03) | (0.02) | (0.02) |
| Wave=11 | 0.00 | 0.00 | 0.00 | 0.00 |
|  | (.) | (.) | (.) | (.) |
| Wave=22 | -0.01 | -0.02 | 0.04^***^ | 0.02^**^ |
|  | (0.01) | (0.01) | (0.01) | (0.01) |
| Interest in EU elections |  | 0.03 |  | 0.06^***^ |
|  |  | (0.02) |  | (0.02) |
| Interest in general politics |  | 0.03 |  | -0.04 |
|  |  | (0.03) |  | (0.02) |
| Support EU border protection |  | 0.10^***^ |  | -0.02 |
|  |  | (0.01) |  | (0.01) |
| Left-Right orientation |  | -0.09^**^ |  | -0.06^*^ |
|  |  | (0.03) |  | (0.03) |
| Support defence spending |  | 0.01 |  | 0.08^***^ |
|  |  | (0.01) |  | (0.01) |
| Prioritise climate protection |  | 0.05^**^ |  | 0.03^*^ |
|  |  | (0.02) |  | (0.01) |
| Constant | 0.43^***^ | 0.37^***^ | 0.15^***^ | 0.16^***^ |
|  | (0.03) | (0.04) | (0.03) | (0.03) |
| Observations | 14127 | 14127 | 14141 | 14141 |
| Respondents | 8865 | 8865 | 8873 | 8873 |
| R² within | 0.0404 | 0.0583 | 0.0523 | 0.0655 |
| R² between | 0.236 | 0.269 | 0.455 | 0.450 |
| R² overall | 0.210 | 0.241 | 0.412 | 0.409 |

# A.5 Robustness Checks

## A.5.1 PSDE

### A.5.1.1 Models without wave fixed effects (one-way fixed effects)

The exclusion of a wave fixed effect does not alter the direction of any effect for the variables of theoretical interest. However, the effect of immigration support becomes significant in all models.

Table 19: Results from one-way fixed effects regressions in PSDE

|  | **(1)** | **(2)** | **(3)** | **(4)** |
| --- | --- | --- | --- | --- |
|  | **European social citizenship support** | **European social citizenship support** | **Welfare European solidarity** | **Welfare European solidarity** |
|  | b | b | b | b |
| German Identification | 0.04 | 0.04 | 0.10* | 0.11* |
|  | (0.05) | (0.04) | (0.05) | (0.05) |
| European Identification | 0.01 | -0.00 | 0.08 | 0.07 |
|  | (0.05) | (0.05) | (0.04) | (0.04) |
| Economic Pol. Orientation | -0.03 | -0.04 | 0.07 | 0.06 |
|  | (0.05) | (0.04) | (0.05) | (0.05) |
| Immigration support | 0.16*** | 0.15*** | 0.10** | 0.08* |
|  | (0.03) | (0.03) | (0.03) | (0.03) |
| Personal income | -0.03 | -0.01 | -0.13 | -0.11 |
|  | (0.09) | (0.09) | (0.12) | (0.12) |
| Left-Right orientation |  | -0.20* |  | -0.21* |
|  |  | (0.09) |  | (0.09) |
| Political Trust |  | 0.14* |  | 0.16** |
|  |  | (0.06) |  | (0.06) |
| General Trust |  | 0.11* |  | 0.05 |
|  |  | (0.05) |  | (0.05) |
| Constant | 0.28*** | 0.27*** | 0.42*** | 0.44*** |
|  | (0.05) | (0.07) | (0.06) | (0.08) |
| Observations | 2736 | 2736 | 2733 | 2733 |
| Respondents | 1455 | 1455 | 1455 | 1455 |
| R² Within | 0.0222 | 0.0367 | 0.0223 | 0.0351 |
| R² Between | 0.361 | 0.440 | 0.346 | 0.405 |
| R² Overall | 0.276 | 0.366 | 0.271 | 0.327 |

^*^ *p* < 0.05, ^**^ *p* < 0.01, ^***^ *p* < 0.001

### A.5.1.2 Models with weights applied

The inclusion of weights to account for differential attrition does not alter the direction of any effect for the variables of theoretical interest. The effect of immigration support becomes insignificant in model 3, likely due to the reduced power of the models including weights.

Table 20: Results from weighted regressions in PSDE

|  | **(1)** | **(2)** | **(3)** | **(4)** |
| --- | --- | --- | --- | --- |
|  | **European social citizenship support** | **European social citizenship support** | **Welfare European solidarity** | **Welfare European solidarity** |
|  | b | b | b | b |
| German Identification | 0.04 | 0.04 | 0.11^*^ | 0.11^*^ |
|  | (0.04) | (0.04) | (0.05) | (0.05) |
| European Identification | -0.00 | -0.01 | 0.07 | 0.06 |
|  | (0.05) | (0.05) | (0.04) | (0.04) |
| Economic Pol. Orientation | -0.04 | -0.04 | 0.06 | 0.05 |
|  | (0.04) | (0.04) | (0.05) | (0.05) |
| Immigration support | 0.11^**^ | 0.10^**^ | 0.07 | 0.06 |
|  | (0.03) | (0.03) | (0.04) | (0.04) |
| Personal Income | 0.05 | 0.06 | -0.09 | -0.08 |
|  | (0.09) | (0.09) | (0.13) | (0.12) |
| Wave=1 | 0.00 | 0.00 | 0.00 | 0.00 |
|  | (.) | (.) | (.) | (.) |
| Wave=2 | -0.06^***^ | -0.06^***^ | -0.03^***^ | -0.02^**^ |
|  | (0.01) | (0.01) | (0.01) | (0.01) |
| Left-Right orientation |  | -0.15 |  | -0.17 |
|  |  | (0.09) |  | (0.09) |
| Political Trust |  | 0.06 |  | 0.13^*^ |
|  |  | (0.06) |  | (0.06) |
| General Trust |  | 0.11^*^ |  | 0.05 |
|  |  | (0.05) |  | (0.05) |
| Constant | 0.31^***^ | 0.31^***^ | 0.44^***^ | 0.44^***^ |
|  | (0.05) | (0.07) | (0.06) | (0.08) |
| Observations | 2736 | 2736 | 2733 | 2733 |
| Respondents | 1455 | 1455 | 1455 | 1455 |
| R² Within | 0.0605 | 0.0686 | 0.0303 | 0.0389 |
| R² Between | 0.255 | 0.405 | 0.317 | 0.396 |
| R² Overall | 0.163 | 0.322 | 0.240 | 0.318 |

^*^ *p* < 0.05, ^**^ *p* < 0.01, ^***^ *p* < 0.001

### A.5.1.3 Models with east/west Germany interactions

The following models allow the effects to differ between east and west Germany. There is only one notable moderation effect: For those in East Germany, there is no significant effect of change in support of relaxing immigration restrictions on support for European Social Citizenship. In contrast, the effect is amongst the strongest predictors of change in support for European Social Citizenship in West Germany.

Table 21: Results from Models with east/west Germany interactions

|  | **(1)** | **(2)** | **(3)** | **(4)** |
| --- | --- | --- | --- | --- |
|  | **European social citizenship support** | **European social citizenship support** | **Welfare European solidarity** | **Welfare European solidarity** |
|  | b | b | b | b |
| German Identification | 0.12 | 0.12 | -0.04 | -0.03 |
|  | (0.11) | (0.11) | (0.12) | (0.11) |
| European Identification | -0.17 | -0.17 | -0.04 | -0.04 |
|  | (0.13) | (0.12) | (0.08) | (0.08) |
| Economic Pol. Orientation | 0.13 | 0.13 | 0.04 | 0.01 |
|  | (0.14) | (0.13) | (0.14) | (0.14) |
| Immigration support | -0.06 | -0.07 | 0.12 | 0.12 |
|  | (0.07) | (0.07) | (0.07) | (0.07) |
| Personal Income | -0.17 | -0.16 | 0.16 | 0.21 |
|  | (0.24) | (0.23) | (0.38) | (0.38) |
| Wave=2 | -0.06^**^ | -0.06^**^ | -0.02 | -0.02 |
|  | (0.02) | (0.02) | (0.02) | (0.02) |
| West=1 # German Identification | -0.10 | -0.09 | 0.17 | 0.16 |
|  | (0.12) | (0.12) | (0.13) | (0.13) |
| West=1 # European Identification | 0.21 | 0.20 | 0.15 | 0.13 |
|  | (0.14) | (0.13) | (0.09) | (0.09) |
| West=1 # Economic Pol. Orientation | -0.18 | -0.19 | 0.04 | 0.06 |
|  | (0.14) | (0.14) | (0.15) | (0.15) |
| West=1 # Immigration support | 0.21^**^ | 0.21^**^ | -0.05 | -0.06 |
|  | (0.08) | (0.08) | (0.08) | (0.08) |
| West=1 # Personal Income | 0.22 | 0.22 | -0.29 | -0.33 |
|  | (0.26) | (0.25) | (0.40) | (0.39) |
| Wave=1 # West=1 | -0.00 | -0.00 | 0.00 | 0.00 |
|  | (0.02) | (0.02) | (0.02) | (0.02) |
| Wave=2 # West=1 | 0.00 | 0.00 | 0.00 | 0.00 |
|  | (.) | (.) | (.) | (.) |
| Left-Right orientation |  | -0.13 |  | -0.31 |
|  |  | (0.18) |  | (0.18) |
| Political Trust |  | 0.13 |  | 0.03 |
|  |  | (0.19) |  | (0.16) |
| General Trust |  | 0.22^*^ |  | -0.10 |
|  |  | (0.11) |  | (0.12) |
| West=1 # Left-Right orientation |  | -0.03 |  | 0.15 |
|  |  | (0.21) |  | (0.21) |
| West=1 # Political Trust |  | -0.07 |  | 0.13 |
|  |  | (0.20) |  | (0.17) |
| West=1 # General Trust |  | -0.13 |  | 0.19 |
|  |  | (0.12) |  | (0.13) |
| Constant | 0.31^***^ | 0.30^***^ | 0.43^***^ | 0.44^***^ |
|  | (0.05) | (0.07) | (0.06) | (0.08) |
| Observations | 2736 | 2736 | 2733 | 2733 |
| Respondents | 1455 | 1455 | 1455 | 1455 |
| R² Within | 0.0693 | 0.0793 | 0.0349 | 0.0473 |
| R² Between | 0.0720 | 0.358 | 0.140 | 0.130 |
| R² Overall | 0.0682 | 0.291 | 0.118 | 0.112 |

^*^ *p* < 0.05, ^**^ *p* < 0.01, ^***^ *p* < 0.001. Note: Base levels omitted from display for brevity.

## A.5.2 GLES data

### A.5.2.1 Models without wave fixed effects (one-way fixed effects)

Results from models without the fixed effect for the survey wave differ only in minor ways from models with two-way fixed effects. The estimated effect of regional household income becomes larger and significant in three of four models.

Table 22: Results from one-way fixed effects regressions in GLES

|  | **(1)** | **(2)** | **(3)** | **(4)** |
| --- | --- | --- | --- | --- |
|  | **Territorial European solidarity** | **Territorial European solidarity** | **Fiscal European solidarity** | **Fiscal European solidarity** |
|  | b | b | b | b |
| German Identification | -0.03 | -0.04 | -0.02 | -0.02 |
|  | (0.02) | (0.02) | (0.02) | (0.02) |
| European Identification | 0.07^***^ | 0.06^***^ | 0.09^***^ | 0.08^***^ |
|  | (0.02) | (0.02) | (0.02) | (0.02) |
| Economic Pol. Orientation | 0.14^***^ | 0.12^***^ | 0.12^***^ | 0.12^***^ |
|  | (0.03) | (0.03) | (0.02) | (0.02) |
| Transnational Pol. Orientation | 0.17^***^ | 0.15^***^ | 0.26^***^ | 0.23^***^ |
|  | (0.03) | (0.03) | (0.03) | (0.03) |
| Personal economic situation | 0.01 | 0.01 | 0.02 | 0.01 |
|  | (0.02) | (0.02) | (0.02) | (0.02) |
| Sociotropic economic situation | 0.04^*^ | 0.03 | 0.05^**^ | 0.05^**^ |
|  | (0.02) | (0.02) | (0.02) | (0.02) |
| Regional household income | -0.02 | -0.02^*^ | 0.03^***^ | 0.02^**^ |
|  | (0.01) | (0.01) | (0.01) | (0.01) |
| Regional mortality | -0.07^*^ | -0.06^*^ | 0.02 | 0.01 |
|  | (0.03) | (0.03) | (0.03) | (0.02) |
| Regional refugee inflow | 0.04 | 0.04 | -0.03 | -0.03 |
|  | (0.03) | (0.03) | (0.02) | (0.02) |
| Interest in EU elections |  | 0.04 |  | 0.06^**^ |
|  |  | (0.02) |  | (0.02) |
| Interest in general politics |  | 0.02 |  | -0.04 |
|  |  | (0.03) |  | (0.02) |
| Support EU border protection |  | 0.10^***^ |  | -0.02 |
|  |  | (0.01) |  | (0.01) |
| Left-Right orientation |  | -0.09^**^ |  | -0.06^*^ |
|  |  | (0.03) |  | (0.03) |
| Support defence spending |  | 0.00 |  | 0.09^***^ |
|  |  | (0.01) |  | (0.01) |
| Prioritise climate protection |  | 0.05^**^ |  | 0.03 |
|  |  | (0.02) |  | (0.01) |
| Constant | 0.43^***^ | 0.37^***^ | 0.16^***^ | 0.17^***^ |
|  | (0.03) | (0.04) | (0.03) | (0.03) |
| Observations | 14127 | 14127 | 14141 | 14141 |
| Respondents | 8865 | 8865 | 8873 | 8873 |
| R² Within | 0.0402 | 0.0578 | 0.0491 | 0.0643 |
| R² Between | 0.235 | 0.268 | 0.455 | 0.448 |
| R² Overall | 0.209 | 0.240 | 0.412 | 0.407 |

^*^ *p* < 0.05, ^**^ *p* < 0.01, ^***^ *p* < 0.001

### A.5.2.2 Models with weights applied

Using weights reduces the sample size, as some respondents did not receive weights in the first wave of the panel survey. The effect of German identification in model 2 becomes insignificant, but does not change in magnitude, indicating that this change may be due to the reduced sample size. The effect of regional mortality loses its significance in model 1.

Table 23: Results from weighted regressions in GLES

|  | **(1)** | **(2)** | **(3)** | **(4)** |
| --- | --- | --- | --- | --- |
|  | **Territorial European solidarity** | **Territorial European solidarity** | **Fiscal European solidarity** | **Fiscal European solidarity** |
|  | b | b | b | b |
| German Identification | -0.03 | -0.04 | -0.01 | -0.02 |
|  | (0.02) | (0.02) | (0.02) | (0.02) |
| European Identification | 0.07^***^ | 0.07^**^ | 0.07^***^ | 0.09^***^ |
|  | (0.02) | (0.02) | (0.02) | (0.02) |
| Economic Pol. Orientation | 0.12^***^ | 0.10^***^ | 0.09^**^ | 0.09^***^ |
|  | (0.03) | (0.03) | (0.03) | (0.03) |
| Transnational Pol. Orientation | 0.14^***^ | 0.13^***^ | 0.21^***^ | 0.23^***^ |
|  | (0.03) | (0.03) | (0.02) | (0.03) |
| Personal economic situation | 0.01 | 0.01 | -0.01 | 0.01 |
|  | (0.02) | (0.02) | (0.02) | (0.02) |
| Sociotropic economic situation | 0.03 | 0.02 | 0.04^*^ | 0.06^**^ |
|  | (0.02) | (0.02) | (0.02) | (0.02) |
| Regional household income | -0.01 | -0.00 | 0.02 | 0.01 |
|  | (0.01) | (0.01) | (0.01) | (0.01) |
| Regional mortality | -0.06 | -0.05 | -0.02 | -0.01 |
|  | (0.03) | (0.03) | (0.03) | (0.03) |
| Regional refugee inflow | 0.04 | 0.04 | -0.03 | -0.03 |
|  | (0.03) | (0.03) | (0.03) | (0.03) |
| Wave=11 | 0.00 | 0.00 | 0.00 | 0.00 |
|  | (.) | (.) | (.) | (.) |
| Wave=22 | -0.01 | -0.02 | 0.03^**^ | 0.03^*^ |
|  | (0.01) | (0.01) | (0.01) | (0.01) |
| Interest in EU elections |  | 0.03 |  | 0.07^***^ |
|  |  | (0.02) |  | (0.02) |
| Interest in general politics |  | 0.03 |  | -0.04 |
|  |  | (0.03) |  | (0.03) |
| Support EU border protection |  | 0.11^***^ |  | -0.01 |
|  |  | (0.02) |  | (0.01) |
| Left-Right orientation |  | -0.10^**^ |  | -0.07^*^ |
|  |  | (0.03) |  | (0.03) |
| Support defence spending |  | 0.00 |  | 0.08^***^ |
|  |  | (0.02) |  | (0.01) |
| Prioritise climate protection |  | 0.04 |  | 0.02 |
|  |  | (0.02) |  | (0.02) |
| Constant | 0.46^***^ | 0.40^***^ | 0.30^***^ | 0.17^***^ |
|  | (0.03) | (0.04) | (0.03) | (0.04) |
| Observations | 11854 | 11854 | 10642 | 11866 |
| Respondents | 6830 | 6830 | 6328 | 6837 |
| R² Within | 0.0346 | 0.0539 | 0.0463 | 0.0654 |
| R² Between | 0.249 | 0.280 | 0.288 | 0.473 |
| R² Overall | 0.210 | 0.239 | 0.250 | 0.416 |

^*^ *p* < 0.05, ^**^ *p* < 0.01, ^***^ *p* < 0.001

### A.5.2.3 Models with east/west Germany interactions

The following models allow the effects to differ between east and west Germany. They show no significant interaction effects, indicating no effect heterogeneity between east and west Germany.

Table 24: Results from Models with east/west Germany interactions

|  | **(1)** | | **(2)** | | **(3)** | | **(4)** |
| --- | --- | --- | --- | --- | --- | --- | --- |
|  | **Territorial European solidarity** | | **Territorial European solidarity** | | **Fiscal European solidarity** | | **Fiscal European solidarity** |
|  | b | | b | | b | | b |
| German Identification | 0.00 | | -0.02 | | 0.01 | | 0.01 |
|  | (0.04) | | (0.04) | | (0.04) | | (0.04) |
| European Identification | 0.09^*^ | | 0.07 | | 0.15^***^ | | 0.14^***^ |
|  | (0.04) | | (0.04) | | (0.04) | | (0.04) |
| Economic Pol. Orientation | 0.17^**^ | | 0.13^*^ | | 0.16^***^ | | 0.14^**^ |
|  | (0.06) | | (0.06) | | (0.05) | | (0.05) |
| Transnational Political Orientation | 0.15^*^ | | 0.12^*^ | | 0.25^***^ | | 0.22^***^ |
|  | (0.06) | | (0.06) | | (0.05) | | (0.05) |
| Personal economic situation | -0.05 | | -0.06 | | 0.04 | | 0.03 |
|  | (0.04) | | (0.04) | | (0.03) | | (0.03) |
| Sociotropic economic situation | 0.02 | | 0.00 | | 0.06 | | 0.06 |
|  | (0.04) | | (0.04) | | (0.03) | | (0.03) |
| Regional household income | -0.07 | | -0.06 | | 0.15^*^ | | 0.14 |
|  | (0.08) | | (0.08) | | (0.07) | | (0.07) |
| Regional mortality | -0.06 | | -0.05 | | 0.04 | | 0.03 |
|  | (0.04) | | (0.04) | | (0.03) | | (0.03) |
| Regional refugee inflow | 0.08 | | 0.09 | | -0.04 | | -0.03 |
|  | (0.05) | | (0.05) | | (0.04) | | (0.04) |
| Wave=22 | 0.00 | | -0.02 | | -0.11 | | -0.12 |
|  | (0.06) | | (0.06) | | (0.06) | | (0.06) |
| West=1 # German Identification | -0.04 | | -0.03 | | -0.04 | | -0.04 |
|  | (0.05) | | (0.05) | | (0.04) | | (0.04) |
| West=1 # European Identification | -0.02 | | -0.02 | | -0.08 | | -0.08 |
|  | (0.04) | | (0.04) | | (0.04) | | (0.04) |
| West=1 # Economic Pol. Orientation | -0.04 | | -0.02 | | -0.05 | | -0.03 |
|  | (0.07) | | (0.06) | | (0.06) | | (0.06) |
| West=1 # Transnational Political Orientation | 0.03 | | 0.04 | | 0.01 | | 0.01 |
|  | (0.07) | | (0.07) | | (0.06) | | (0.06) |
| West=1 # Personal economic situation | 0.08 | | 0.09 | | -0.03 | | -0.02 |
|  | (0.05) | | (0.05) | | (0.04) | | (0.04) |
| West=1 # Sociotropic economic situation | 0.01 | | 0.03 | | 0.01 | | 0.00 |
|  | (0.04) | | (0.04) | | (0.04) | | (0.04) |
| West=1 # Regional household income | 0.08 | | 0.06 | | -0.13 | | -0.12 |
|  | (0.09) | | (0.08) | | (0.08) | | (0.08) |
| West=1 # Regional mortality | 0.01 | | 0.01 | | -0.04 | | -0.02 |
|  | (0.06) | | (0.06) | | (0.06) | | (0.06) |
| West=1 # Regional refugee inflow | -0.00 | | -0.01 | | 0.03 | | 0.01 |
|  | (0.07) | | (0.07) | | (0.06) | | (0.06) |
| Wave=11 # West=1 | 0.03 | | 0.01 | | -0.13^*^ | | -0.13^*^ |
|  | (0.06) | | (0.06) | | (0.06) | | (0.06) |
| Wave=22 # West=1 | 0.00 | | 0.00 | | 0.00 | | 0.00 |
|  | (.) | | (.) | | (.) | | (.) |
| Interest in EU elections |  | | 0.06 | |  | | 0.05 |
|  |  | | (0.04) | |  | | (0.03) |
| Interest in general politics |  | | -0.01 | |  | | -0.08 |
|  |  | | (0.05) | |  | | (0.05) |
| Support EU border protection |  | | 0.14^***^ | |  | | -0.05^*^ |
|  |  | (0.03) | |  | | (0.02) | |
| Left-Right orientation |  | -0.13^*^ | |  | | -0.03 | |
|  |  | (0.06) | |  | | (0.05) | |
| Support defence spending |  | 0.04 | |  | | 0.07^**^ | |
|  |  | (0.03) | |  | | (0.02) | |
| Prioritise climate protection |  | 0.09^**^ | |  | | 0.05 | |
|  |  | (0.03) | |  | | (0.03) | |
| West=1 # Interest in EU elections |  | -0.03 | |  | | 0.02 | |
|  |  | (0.05) | |  | | (0.04) | |
| West=1 # Interest in general politics |  | 0.05 | |  | | 0.05 | |
|  |  | (0.06) | |  | | (0.06) | |
| West=1 # Support EU border protection |  | -0.06 | |  | | 0.05 | |
|  |  | (0.03) | |  | | (0.03) | |
| West=1 # Left-Right orientation |  | 0.05 | |  | | -0.03 | |
|  |  | (0.07) | |  | | (0.06) | |
| West=1 # Support defence spending |  | -0.04 | |  | | 0.00 | |
|  |  | (0.03) | |  | | (0.03) | |
| West=1 # Prioritise climate protection |  | -0.05 | |  | | -0.03 | |
|  |  | (0.04) | |  | | (0.03) | |
| Constant | 0.40^***^ | 0.37^***^ | | 0.25^***^ | | 0.25^***^ | |
|  | (0.05) | (0.06) | | (0.05) | | (0.05) | |
| Observations | 14120 | 14120 | | 14134 | | 14134 | |
| Respondents | 8858 | 8858 | | 8866 | | 8866 | |
| R² Within | 0.0424 | 0.0623 | | 0.0559 | | 0.0699 | |
| R² Between | 0.204 | 0.250 | | 0.207 | | 0.300 | |
| R² Overall | 0.185 | 0.227 | | 0.193 | | 0.281 | |

^*^ *p* < 0.05, ^**^ *p* < 0.01, ^***^ *p* < 0.001

### A.5.2.3 Models with GDP per capita instead of disposable income

The following models estimate the crisis impact of economic crisis using regional GDP per capita instead of regional household income. Differences in the change of GDP per capita at the regional level are significantly associated with territorial solidarity, however with a small effect size.

Table 25: Regression Results from Model with GDP per Capita

|  | **(1)** | **(2)** | **(3)** | **(4)** |
| --- | --- | --- | --- | --- |
|  | **Territorial solidarity** | **Territorial solidarity** | **Fiscal solidarity** | **Fiscal solidarity** |
|  | b/se | b/se | b/se | b/se |
| German Identification | -0.03 | -0.04* | -0.02 | -0.02 |
|  | (0.02) | (0.02) | (0.02) | (0.02) |
| European Identification | 0.07*** | 0.06** | 0.09*** | 0.08*** |
|  | (0.02) | (0.02) | (0.02) | (0.02) |
| Economic Pol. Orientation | 0.14*** | 0.12*** | 0.12*** | 0.12*** |
|  | (0.03) | (0.03) | (0.02) | (0.02) |
| Transnational Political Orientation | 0.17*** | 0.15*** | 0.25*** | 0.23*** |
|  | (0.03) | (0.03) | (0.03) | (0.03) |
| Personal economic situation | 0.01 | 0.01 | 0.02 | 0.01 |
|  | (0.02) | (0.02) | (0.02) | (0.02) |
| Sociotropic economic situation | 0.03 | 0.02 | 0.06*** | 0.06*** |
|  | (0.02) | (0.02) | (0.02) | (0.02) |
| Regional GDP per capita | -0.03* | -0.03* | -0.01 | -0.02 |
|  | (0.01) | (0.01) | (0.01) | (0.01) |
| Regional mortality | -0.06* | -0.06* | -0.00 | 0.00 |
|  | (0.03) | (0.03) | (0.02) | (0.02) |
| Regional refugee inflow | 0.05 | 0.05* | -0.03 | -0.03 |
|  | (0.03) | (0.03) | (0.02) | (0.02) |
| Wave=11 | 0.00 | 0.00 | 0.00 | 0.00 |
|  | (.) | (.) | (.) | (.) |
| Wave=22 | -0.01 | -0.01 | 0.04*** | 0.03*** |
|  | (0.01) | (0.01) | (0.01) | (0.01) |
| Interest in EU elections |  | 0.04 |  | 0.06*** |
|  |  | (0.02) |  | (0.02) |
| Interest in general politics |  | 0.03 |  | -0.04 |
|  |  | (0.03) |  | (0.02) |
| Support EU border protection |  | 0.10*** |  | -0.02 |
|  |  | (0.01) |  | (0.01) |
| Left-Right orientation |  | -0.09** |  | -0.06* |
|  |  | (0.03) |  | (0.03) |
| Support defence spending |  | 0.01 |  | 0.08*** |
|  |  | (0.01) |  | (0.01) |
| Prioritise climate protection |  | 0.05** |  | 0.03* |
|  |  | (0.02) |  | (0.01) |
| Constant | 0.43*** | 0.37*** | 0.15*** | 0.16*** |
|  | (0.03) | (0.04) | (0.03) | (0.03) |
| Observations | 14127 | 14127 | 14141 | 14141 |
| Respondents | 8865 | 8865 | 8873 | 8873 |
| R² Within | 0.0412 | 0.0590 | 0.0526 | 0.0658 |
| R² Between | 0.234 | 0.267 | 0.455 | 0.450 |
| R² Overall | 0.209 | 0.240 | 0.412 | 0.409 |

^*^ *p* < 0.05, ^**^ *p* < 0.01, ^***^ *p* < 0.001

### A.5.2.4 Models with Alternative Operationalisations of Transnational Orientation

The models in the following tables make use of the three individual items constituting the index of transnational political orientation. These ask about support for immigration policy (immigration), support for further European integration (EU integration), and whether foreigners in Germany should fully assimilate or be allowed to maintain their culture (cultural diversity). The findings largely confirm the results based on the index of transnational political orientation: increases in support for all three items are significantly associated with territorial and fiscal solidarity, except for the cultural diversity item in model 3 for territorial solidarity.

Table 26: Models with Alternative Operationalisations of Transnational Orientation – Territorial Solidarity

|  | **Territorial Solidarity** | | |
| --- | --- | --- | --- |
|  | **(1)** | **(2)** | **(3)** |
|  | b/se | b/se | b/se |
| German Identification | -0.03 | -0.03 | -0.03 |
|  | (0.02) | (0.02) | (0.02) |
| European Identification | 0.08^***^ | 0.06^***^ | 0.09^***^ |
|  | (0.02) | (0.02) | (0.02) |
| Economic Pol. Orientation | 0.14^***^ | 0.14^***^ | 0.14^***^ |
|  | (0.03) | (0.03) | (0.03) |
| Immigration support | 0.05^**^ |  |  |
|  | (0.02) |  |  |
| EU integration |  | 0.10^***^ |  |
|  |  | (0.02) |  |
| Cultural Diversity |  |  | 0.02 |
|  |  |  | (0.02) |
| Personal Econ. Situation | 0.01 | 0.01 | 0.01 |
|  | (0.02) | (0.02) | (0.02) |
| General Econ. Situation | 0.04^*^ | 0.03 | 0.04^*^ |
|  | (0.02) | (0.02) | (0.02) |
| Regional household income | -0.01 | -0.01 | -0.01 |
|  | (0.01) | (0.01) | (0.01) |
| Regional mortality | -0.06^*^ | -0.06^*^ | -0.06^*^ |
|  | (0.03) | (0.03) | (0.03) |
| Regional refugee inflow | 0.04 | 0.04 | 0.04 |
|  | (0.03) | (0.03) | (0.03) |
| Wave=22 | -0.01 | -0.00 | -0.01 |
|  | (0.01) | (0.01) | (0.01) |
| Constant | 0.47^***^ | 0.44^***^ | 0.48^***^ |
|  | (0.03) | (0.03) | (0.03) |
| Observations | 14107 | 14096 | 14121 |
| Respondents | 8860 | 8858 | 8861 |
| R² Within | 0.0342 | 0.0423 | 0.0326 |
| R² Between | 0.235 | 0.252 | 0.234 |
| R² Overall | 0.204 | 0.221 | 0.200 |

^*^ *p* < 0.05, ^**^ *p* < 0.01, ^***^ *p* < 0.001

Table 27: Models with Alternative Operationalisations of Transnational Orientation – Fiscal Solidarity

|  | **Fiscal Solidarity** | | |
| --- | --- | --- | --- |
|  | **(1)** | **(2)** | **(3)** |
|  | b/se | b/se | b/se |
| German Identification | -0.02 | -0.02 | -0.02 |
|  | (0.02) | (0.02) | (0.02) |
| European Identification | 0.12^***^ | 0.10^***^ | 0.12^***^ |
|  | (0.02) | (0.02) | (0.02) |
| Economic Pol. Orientation | 0.13^***^ | 0.13^***^ | 0.13^***^ |
|  | (0.03) | (0.02) | (0.02) |
| Immigration support | 0.10^***^ |  |  |
|  | (0.02) |  |  |
| EU integration |  | 0.12^***^ |  |
|  |  | (0.01) |  |
| Cultural Diversity |  |  | 0.07^***^ |
|  |  |  | (0.02) |
| Personal Econ. Situation | 0.02 | 0.02 | 0.02 |
|  | (0.02) | (0.02) | (0.02) |
| General Econ. Situation | 0.07^***^ | 0.06^***^ | 0.07^***^ |
|  | (0.02) | (0.02) | (0.02) |
| Regional household income | 0.00 | 0.01 | 0.00 |
|  | (0.01) | (0.01) | (0.01) |
| Regional mortality | -0.01 | -0.00 | -0.01 |
|  | (0.03) | (0.03) | (0.03) |
| Regional refugee inflow | -0.03 | -0.04 | -0.03 |
|  | (0.02) | (0.02) | (0.02) |
| Wave=11 | 0.00 | 0.00 | 0.00 |
|  | (.) | (.) | (.) |
| Wave=22 | 0.04^***^ | 0.04^***^ | 0.04^***^ |
|  | (0.01) | (0.01) | (0.01) |
| Constant | 0.20^***^ | 0.19^***^ | 0.21^***^ |
|  | (0.02) | (0.02) | (0.02) |
| Observations | 14089 | 14078 | 14103 |
| Respondents | 8857 | 8855 | 8858 |
| R² Within | 0.0386 | 0.0458 | 0.0341 |
| R² Between | 0.424 | 0.402 | 0.393 |
| R² Overall | 0.381 | 0.363 | 0.350 |

^*^ *p* < 0.05, ^**^ *p* < 0.01, ^***^ *p* < 0.001

# A.6 Paired t-Tests of Change in European Solidarity

## A.6.1 PSDE

Paired t-Tests for both European social citizenship support and welfare solidarity in the PSDE data show significant change between the two waves.

Table 28: Paired t-test for social citizenship and welfare solidarity in PSDE

**Paired t test : Social citizenship Wave 1 Social citizenship Wave 2**

|  | Obs | Mean1 | Mean2 | Diff | St Err | t value | p value |
| --- | --- | --- | --- | --- | --- | --- | --- |
| ESCSW1 – ESCSW2 | 1515 | 0.373 | 0.312 | 0.061 | 0.007 | 8.35 | < 0.01 |

**Paired t test : Welfare Solidarity Wave 1 Welfare Solidarity Wave 2**

|  | Obs | Mean1 | Mean2 | Diff | St Err | t value | p value |
| --- | --- | --- | --- | --- | --- | --- | --- |
| WELSW1 – WELSW2 | 1505 | 0.599 | 0.564 | 0.035 | 0.007 | 4.65 | < 0.01 |

### A.6.2 GLES

Paired t-Tests for both territorial and fiscal solidarity in the GLES data show significant change between the two waves.

Table 29: Paired t-test for territorial and fiscal solidarity in GLES

**Paired t test : Territorial Solidarity Wave 11 Territorial Solidarity Wave 22**

|  | Obs | Mean1 | Mean2 | Diff | St Err | t value | p value |
| --- | --- | --- | --- | --- | --- | --- | --- |
| TerSolW11 – TerSolW22 | 6137 | 0.62 | 0.589 | 0.031 | 0.003 | 9.5 | < 0.01 |

**Paired t test : Fiscal Solidarity Wave 11 Fiscal Solidarity Wave 22**

|  | Obs | Mean1 | Mean2 | Diff | St Err | t value | p value |
| --- | --- | --- | --- | --- | --- | --- | --- |
| FisSolW11 – FisSolW22 | 6145 | 0.415 | 0.426 | -0.011 | 0.003 | -3.85 | < 0.01 |

# A.7 Multinomial Logistic Regression

## A.7.1 Stability Across Initial Levels of Political Orientation

Here, I first present the plots of average marginal effects on the probabilities to show a decrease, stability or an increase in each type of European solidarity for economic and transnational political orientation. I then report the tables of multinomial logistic regression results.

Figure 1: Predicted Probabilities of change/stability in European Solidarity in the PSDE (top row) and GLES (bottom row) data by economic political orientation

| 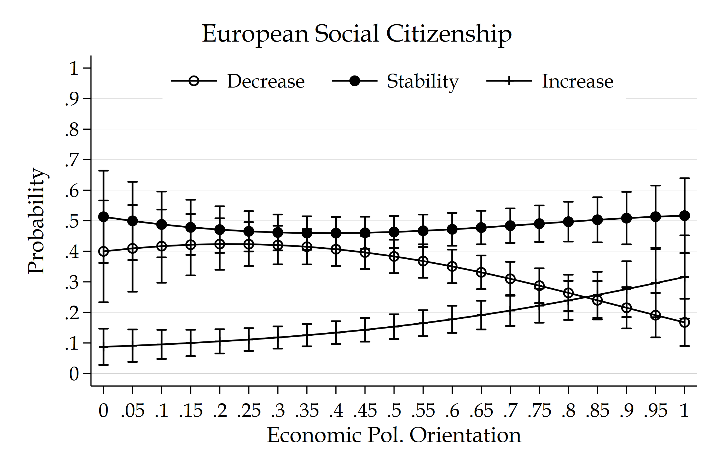 | 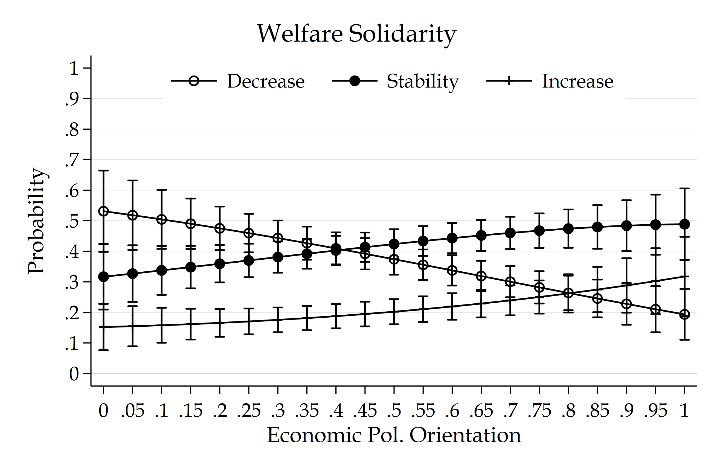 |
| --- | --- |
| 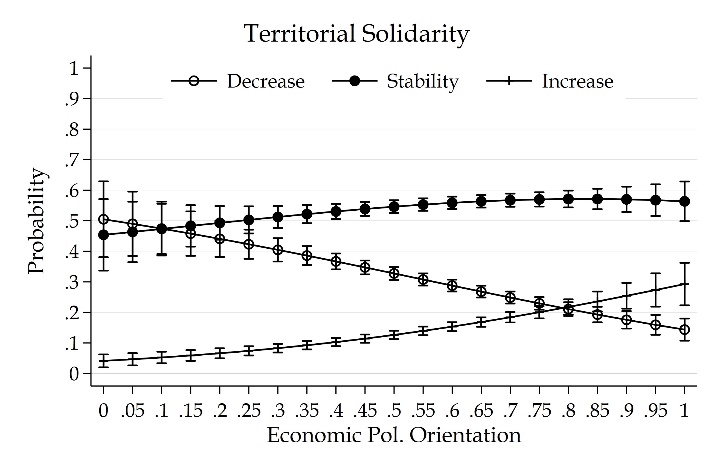 | 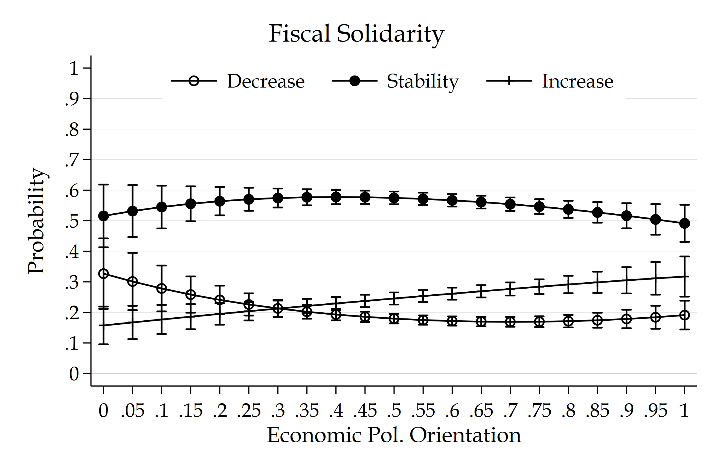 |

Figure 2: Predicted Probabilities of change/stability in European Solidarity in the PSDE (top row) and GLES (bottom row) data by transnational political orientation

| 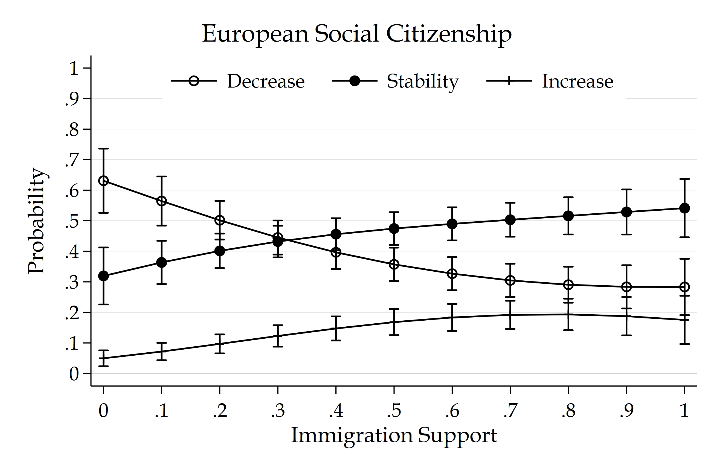 | 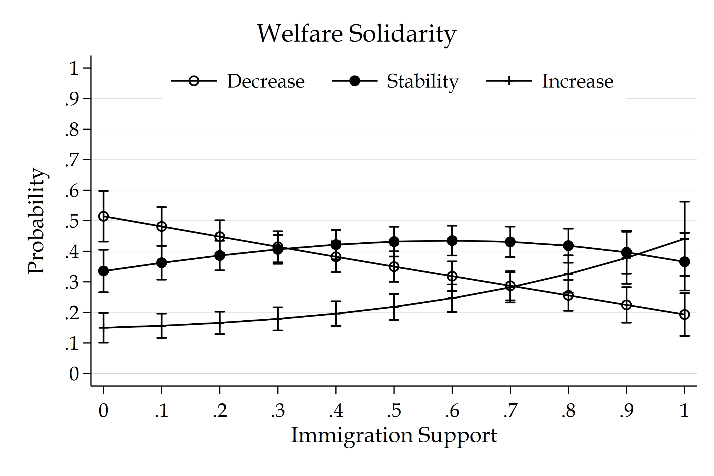 |
| --- | --- |
| 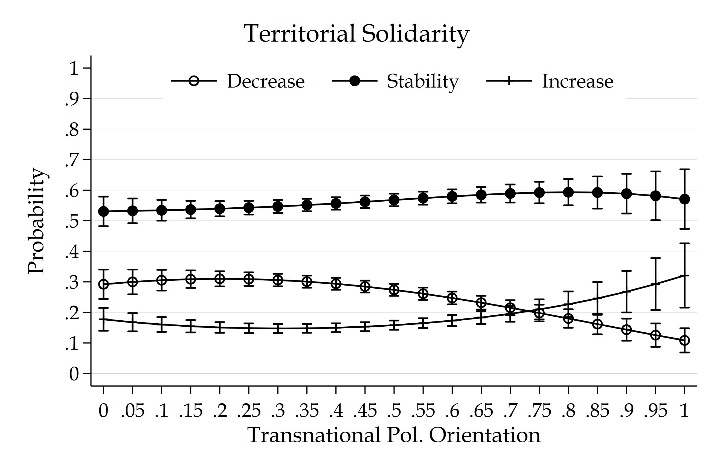 | 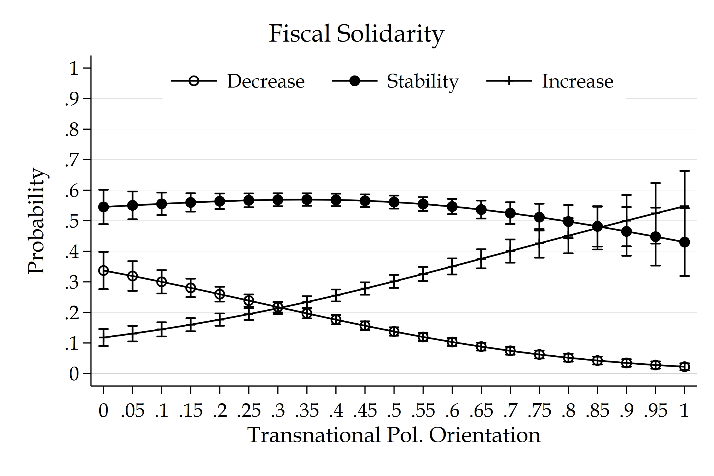 |

## A.7.2 PSDE

Here, I report the regression results tables for the multinomial logistic regression analysis of decrease, stability and increase in European Solidarity.

Table 30: Results Table of multinomial logistic regression with dependent variable indication decrease, stability, and increase in European social citizenship support and welfare solidarity in PSDE. The “No change” category is the reference category.

|  | **(1)** | **(2)** |
| --- | --- | --- |
|  | **European social citizenship support** | **Welfare Solidarity** |
|  | b/se | b/se |
| **Decrease** |  |  |
| Male & Diverse | 1.00 | 1.00 |
|  | (.) | (.) |
| Female | 1.51^*^ | 1.08 |
|  | (0.24) | (0.16) |
| 18-35 years old | 0.99 | 0.72 |
|  | (0.23) | (0.16) |
| 36-55 years old | 1.30 | 0.76 |
|  | (0.24) | (0.13) |
| 56+ years old | 1.00 | 1.00 |
|  | (.) | (.) |
| Low Educated | 1.00 | 1.00 |
|  | (.) | (.) |
| Middle Educated | 1.02 | 0.91 |
|  | (0.21) | (0.17) |
| High Educated | 0.82 | 1.00 |
|  | (0.18) | (0.20) |
| Personal Income | 0.62 | 0.84 |
|  | (0.38) | (0.46) |
| Immigration Support | 0.07^**^ | 0.34 |
|  | (0.07) | (0.26) |
| Immigration Support # Immigration Support | 4.60 | 1.13 |
|  | (4.02) | (0.82) |
| Economic Pol. Orientation | 3.80 | 0.25 |
|  | (5.01) | (0.26) |
| Economic Pol. Orientation # Economic Pol. Orientation | 0.11 | 1.06 |
|  | (0.13) | (1.05) |
| German Identification | 3.36^**^ | 2.52^**^ |
|  | (1.29) | (0.86) |
| European Identification | 0.31^**^ | 0.23^***^ |
|  | (0.11) | (0.08) |
| Political Trust | 0.53 | 0.84 |
|  | (0.21) | (0.29) |
| General Trust | 0.66 | 0.59 |
|  | (0.25) | (0.20) |
| European Social Citizenship Support | 7461427.40^***^ |  |
|  | (9143382.79) |  |
| European Social Citizenship Support # European Social Citizenship Support | 0.00^***^ |  |
|  | (0.00) |  |
| Welfare Solidarity |  | 2163.43^***^ |
|  |  | (2424.54) |
| Welfare Solidarity # Welfare Solidarity |  | 0.02^***^ |
|  |  | (0.02) |
| **No Change (ref.)** |  |  |
| **Increase** |  |  |
| Male & Diverse | 1.00 | 1.00 |
|  | (.) | (.) |
| Female | 0.85 | 0.75 |
|  | (0.15) | (0.12) |
| 18-35 years old | 0.78 | 0.94 |
|  | (0.21) | (0.23) |
| 36-55 years old | 0.86 | 1.07 |
|  | (0.17) | (0.20) |
| 56+ years old | 1.00 | 1.00 |
|  | (.) | (.) |
| Low Educated | 1.00 | 1.00 |
|  | (.) | (.) |
| Middle Educated | 1.36 | 1.24 |
|  | (0.30) | (0.26) |
| High Educated | 1.53 | 1.59^*^ |
|  | (0.38) | (0.36) |
| Personal Income | 0.24 | 0.35 |
|  | (0.18) | (0.23) |
| Immigration Support | 9.97^*^ | 0.31 |
|  | (9.33) | (0.26) |
| Immigration Support # Immigration Support | 0.19 | 7.05^*^ |
|  | (0.17) | (6.06) |
| Economic Pol. Orientation | 4.76 | 0.85 |
|  | (6.41) | (0.97) |
| Economic Pol. Orientation # Economic Pol. Orientation | 0.77 | 1.53 |
|  | (0.97) | (1.69) |
| German Identification | 1.35 | 0.86 |
|  | (0.58) | (0.32) |
| European Identification | 1.66 | 2.36^*^ |
|  | (0.65) | (0.84) |
| Political Trust | 1.43 | 2.22^*^ |
|  | (0.61) | (0.89) |
| General Trust | 1.40 | 0.88 |
|  | (0.59) | (0.33) |
| European Social Citizenship Support | 1.51 |  |
|  | (1.40) |  |
| European Social Citizenship Support # European Social Citizenship Support | 0.02^**^ |  |
|  | (0.02) |  |
| Welfare Solidarity |  | 36.87^***^ |
|  |  | (33.08) |
| Welfare Solidarity # Welfare Solidarity |  | 0.00^***^ |
|  |  | (0.00) |
| Observations | 1350 | 1346 |
| *AIC* | 2043.5 | 2422.9 |
| *BIC* | 2220.6 | 2599.9 |

Exponentiated coefficients

^*^ *p* < 0.05, ^**^ *p* < 0.01, ^***^ *p* < 0.001

## A.7.3 GLES

Here, I report the tables for the multinomial logistic regression analysis of decrease, stability and increase in European Solidarity.

Table 31: Results table of multinomial logistic regression with dependent variable indication decrease, stability, and increase in territorial and fiscal solidarity in GLES. The “No change” category is the reference category.

|  | **(1)** | **(2)** |
| --- | --- | --- |
|  | **Territorial Solidarity** | **Fiscal Solidarity** |
|  | b/se | b/se |
| **Decrease** |  |  |
| Male | 1.00 | 1.00 |
|  | (.) | (.) |
| Female | 1.15^*^ | 1.12 |
|  | (0.08) | (0.08) |
| 20-35 years old | 1.20 | 0.97 |
|  | (0.13) | (0.12) |
| 36-55 years old | 1.00 | 1.00 |
|  | (0.07) | (0.08) |
| 56+ years old | 1.00 | 1.00 |
|  | (.) | (.) |
| Low Educated | 1.00 | 1.00 |
|  | (.) | (.) |
| Middle Educated | 1.18 | 1.22 |
|  | (0.11) | (0.13) |
| High Educated | 1.24^*^ | 1.16 |
|  | (0.12) | (0.12) |
| General Econ. Situation | 0.98 | 0.53^**^ |
|  | (0.18) | (0.11) |
| Transnational Pol. Orientation | 1.70 | 0.30^*^ |
|  | (0.88) | (0.18) |
| Transnational Pol. Orientation # Transnational Pol. Orientation | 0.20^**^ | 0.28 |
|  | (0.11) | (0.19) |
| Economic Pol. Orientation | 0.37 | 0.10^**^ |
|  | (0.31) | (0.09) |
| Economic Pol. Orientation # Economic Pol. Orientation | 0.62 | 6.38^*^ |
|  | (0.44) | (4.79) |
| German Identification | 1.22 | 1.96^***^ |
|  | (0.20) | (0.35) |
| European Identification | 0.45^***^ | 0.43^***^ |
|  | (0.07) | (0.07) |
| Territorial Solidarity | 187.44^***^ |  |
|  | (170.13) |  |
| Territorial Solidarity # Territorial Solidarity | 0.79 |  |
|  | (0.51) |  |
| Fiscal Solidarity |  | 710.39^***^ |
|  |  | (425.13) |
| Fiscal Solidarity # Fiscal Solidarity |  | 0.26^*^ |
|  |  | (0.14) |
| **No Change (ref.)** |  |  |
| **Increase** |  |  |
| Male | 1.00 | 1.00 |
|  | (.) | (.) |
| Female | 0.82^**^ | 0.85^*^ |
|  | (0.06) | (0.06) |
| 20-35 years old | 0.88 | 1.54^***^ |
|  | (0.11) | (0.17) |
| 36-55 years old | 0.72^***^ | 1.04 |
|  | (0.06) | (0.07) |
| 56+ years old | 1.00 | 1.00 |
|  | (.) | (.) |
| Low Educated | 1.00 | 1.00 |
|  | (.) | (.) |
| Middle Educated | 0.96 | 0.84 |
|  | (0.10) | (0.08) |
| High Educated | 0.79^*^ | 0.83^*^ |
|  | (0.08) | (0.08) |
| General Econ. Situation | 1.92^**^ | 2.18^***^ |
|  | (0.40) | (0.42) |
| Transnational Pol. Orientation | 0.29^*^ | 6.51^***^ |
|  | (0.17) | (3.56) |
| Transnational Pol. Orientation # Transnational Pol. Orientation | 5.86^**^ | 0.91 |
|  | (3.90) | (0.56) |
| Economic Pol. Orientation | 7.29^*^ | 1.82 |
|  | (6.35) | (1.43) |
| Economic Pol. Orientation # Economic Pol. Orientation | 0.78 | 1.17 |
|  | (0.60) | (0.81) |
| German Identification | 1.06 | 0.86 |
|  | (0.19) | (0.14) |
| European Identification | 2.60^***^ | 2.80^***^ |
|  | (0.47) | (0.45) |
| Territorial Solidarity | 0.10^***^ |  |
|  | (0.06) |  |
| Territorial Solidarity # Territorial Solidarity | 0.05^***^ |  |
|  | (0.03) |  |
| Fiscal Solidarity |  | 0.35^*^ |
|  |  | (0.17) |
| Fiscal Solidarity # Fiscal Solidarity |  | 0.00^***^ |
|  |  | (0.00) |
| Observations | 6098 | 6106 |
| *AIC* | 10572.4 | 10581.1 |
| *BIC* | 10773.9 | 10782.6 |

Exponentiated coefficients

^*^ *p* < 0.05, ^**^ *p* < 0.01, ^***^ *p* < 0.001

# A.8 Cross-lagged panel models

To test the plausibility of the hypothesised direction of causality between the independent variables and European solidarity (without making claims about causality per se), I estimate cross-lagged panel models for the four dimensions of European solidarity, predicting European solidarity in the second wave of each dataset with itself and the independent variables in the first wave.

## A.8.1 PSDE

I estimate cross-lagged panel models of independent variables and both European social citizenship support and welfare solidarity in waves 1 and 22of the PSDE panel.

The main information from these models is summarised in Table 32 (see Table 33 for the full model): the causal pathway between the independent variables and fiscal and territorial European solidarity run primarily in the expected direction: The coefficient in the hypothesised direction is larger in all cases.

Table 32: Summary of Cross-Lagged Associations in PSDE data

|  | **SEM 1** | |  | **SEM 2** | |
| --- | --- | --- | --- | --- | --- |
|  | **Hypothesised direction  W1-Variables to  W2-European Social Citizenship Support** | **Opposite direction W1-European Social Citizenship Support to W2-Variable** |  | **Hypothesised direction  W1-Variables to  W2- Welfare Solidarity** | **Opposite direction W1- Welfare Solidarity to W2-Variable** |
|  | b/se | b/se |  | b/se | b/se |
| European Social Citizenship Support | 0.46^***^ | 0.47^***^ | Welfare Solidarity | 0.39^***^ | 0.47^***^ |
|  | (0.02) | (0.02) |  | (0.02) | (0.02) |
| German Identification | -0.04 | -0.01 | German Identification | -0.06^*^ | -0.00 |
|  | (0.03) | (0.01) |  | (0.03) | (0.01) |
| European Identification | 0.09^***^ | 0.09^***^ | European Identification | 0.15^***^ | 0.11^***^ |
|  | (0.03) | (0.01) |  | (0.03) | (0.02) |
| Economic Pol. Orientation | 0.15^***^ | 0.10^***^ | Economic Pol. Orientation | 0.20^***^ | 0.04^*^ |
|  | (0.03) | (0.02) |  | (0.03) | (0.02) |
| Immigration Support | 0.17^***^ | 0.15^***^ | Immigration Support | 0.15^***^ | 0.14^***^ |
|  | (0.02) | (0.02) |  | (0.02) | (0.02) |
| Personal Income | 0.04 | -0.01 | Personal Income | 0.02 | 0.00 |
|  | (0.05) | (0.01) |  | (0.05) | (0.01) |
| Constant | -0.06^*^ |  | Constant | 0.10^***^ |  |
|  | (0.03) |  |  | (0.03) |  |

Table 33: Full Result of Cross-Lagged Panel Models in PSDE

| **SEM (1)** | | **SEM (2)** | |
| --- | --- | --- | --- |
|  | b/se |  | b/se |
| 2 European Social Citizenship Support |  | 2 Welfare Solidarity |  |
| European Social Citizenship Support | 0.46^***^ | Welfare Solidarity | 0.39^***^ |
|  | (0.02) |  | (0.02) |
| German Identification | -0.04 | German Identification | -0.06^*^ |
|  | (0.03) |  | (0.03) |
| European Identification | 0.09^***^ | European Identification | 0.15^***^ |
|  | (0.03) |  | (0.03) |
| Economic Pol. Orientation | 0.15^***^ | Economic Pol. Orientation | 0.20^***^ |
|  | (0.03) |  | (0.03) |
| Immigration Support | 0.17^***^ | Immigration Support | 0.15^***^ |
|  | (0.02) |  | (0.02) |
| Personal Income | 0.04 | Personal Income | 0.02 |
|  | (0.05) |  | (0.05) |
| Constant | -0.06^*^ | Constant | 0.10^***^ |
|  | (0.03) |  | (0.03) |
| 2 German Identification |  | 2 German Identification |  |
| European Social Citizenship Support | -0.01 | Welfare Solidarity | -0.00 |
|  | (0.01) |  | (0.01) |
| German Identification | 0.60^***^ | German Identification | 0.60^***^ |
|  | (0.02) |  | (0.02) |
| Constant | 0.28^***^ | Constant | 0.28^***^ |
|  | (0.02) |  | (0.02) |
| 2 European Identification |  | 2 European Identification |  |
| European Social Citizenship Support | 0.09^***^ | Welfare Solidarity | 0.11^***^ |
|  | (0.01) |  | (0.02) |
| European Identification | 0.66^***^ | European Identification | 0.64^***^ |
|  | (0.02) |  | (0.02) |
| Constant | 0.18^***^ | Constant | 0.15^***^ |
|  | (0.01) |  | (0.01) |
| 2 Economic Pol. Orientation |  | 2 Economic Pol. Orientation |  |
| European Social Citizenship Support | 0.10^***^ | Welfare Solidarity | 0.04^*^ |
|  | (0.02) |  | (0.02) |
| Economic Pol. Orientation | 0.62^***^ | Economic Pol. Orientation | 0.65^***^ |
|  | (0.02) |  | (0.02) |
| Immigration Support | 0.04^*^ | Immigration Support | 0.07^***^ |
|  | (0.02) |  | (0.02) |
| Constant | 0.14^***^ | Constant | 0.12^***^ |
|  | (0.01) |  | (0.01) |
| 2 Immigration Support |  | 2 Immigration Support |  |
| European Social Citizenship Support | 0.15^***^ | Welfare Solidarity | 0.14^***^ |
|  | (0.02) |  | (0.02) |
| Economic Pol. Orientation | 0.11^***^ | Economic Pol. Orientation | 0.12^***^ |
|  | (0.03) |  | (0.03) |
| Immigration Support | 0.56^***^ | Immigration Support | 0.58^***^ |
|  | (0.02) |  | (0.02) |
| Constant | 0.02 | Constant | -0.02 |
|  | (0.01) |  | (0.02) |
| 2 Personal Income |  | 2 Personal Income |  |
| European Social Citizenship Support | -0.01 | Welfare Solidarity | 0.00 |
|  | (0.01) |  | (0.01) |
| Personal Income | 0.87^***^ | Personal Income | 0.87^***^ |
|  | (0.01) |  | (0.01) |
| Constant | 0.04^***^ | Constant | 0.04^***^ |
|  | (0.00) |  | (0.01) |
| / |  | / |  |
| mean(European Social Citizenship Support1) | 0.37^***^ | mean(Welfare Solidarity1) | 0.60^***^ |
|  | (0.01) |  | (0.01) |
| mean(German Identification1) | 0.73^***^ | mean(German Identification1) | 0.73^***^ |
|  | (0.01) |  | (0.01) |
| mean(European Identification1) | 0.64^***^ | mean(European Identification1) | 0.64^***^ |
|  | (0.01) |  | (0.01) |
| mean(Economic Pol. Orientation1) | 0.51^***^ | mean(Economic Pol. Orientation1) | 0.51^***^ |
|  | (0.01) |  | (0.01) |
| mean(Immigration Support1) | 0.44^***^ | mean(Immigration Support1) | 0.44^***^ |
|  | (0.01) |  | (0.01) |
| mean(Personal Income1) | 0.26^***^ | mean(Personal Income1) | 0.26^***^ |
|  | (0.00) |  | (0.00) |
| var(e.European Social Citizenship Support2) | 0.06^***^ | var(e.Welfare Solidarity2) | 0.06^***^ |
|  | (0.00) |  | (0.00) |
| var(e.German Identification2) | 0.03^***^ | var(e.German Identification2) | 0.03^***^ |
|  | (0.00) |  | (0.00) |
| var(e.European Identification2) | 0.04^***^ | var(e.European Identification2) | 0.04^***^ |
|  | (0.00) |  | (0.00) |
| var(e.Economic Pol. Orientation2) | 0.03^***^ | var(e.Economic Pol. Orientation2) | 0.03^***^ |
|  | (0.00) |  | (0.00) |
| var(e.Immigration Support2) | 0.05^***^ | var(e.Immigration Support2) | 0.05^***^ |
|  | (0.00) |  | (0.00) |
| var(e.Personal Income2) | 0.01^***^ | var(e.Personal Income2) | 0.01^***^ |
|  | (0.00) |  | (0.00) |
| var(European Social Citizenship Support1) | 0.12^***^ | var(Welfare Solidarity1) | 0.11^***^ |
|  | (0.00) |  | (0.00) |
| var(German Identification1) | 0.06^***^ | var(German Identification1) | 0.06^***^ |
|  | (0.00) |  | (0.00) |
| var(European Identification1) | 0.08^***^ | var(European Identification1) | 0.08^***^ |
|  | (0.00) |  | (0.00) |
| var(Economic Pol. Orientation1) | 0.06^***^ | var(Economic Pol. Orientation1) | 0.06^***^ |
|  | (0.00) |  | (0.00) |
| var(Immigration Support1) | 0.11^***^ | var(Immigration Support1) | 0.11^***^ |
|  | (0.00) |  | (0.00) |
| var(Personal Income1) | 0.02^***^ | var(Personal Income1) | 0.02^***^ |
|  | (0.00) |  | (0.00) |
| cov(e.European Social Citizenship Support2,e.German Identification2) | 0.00 | cov(e.Welfare Solidarity2,e.German Identification2) | 0.00^**^ |
|  | (0.00) |  | (0.00) |
| cov(e.European Social Citizenship Support2,e.European Identification2) | 0.00^**^ | cov(e.Welfare Solidarity2,e.European Identification2) | 0.01^***^ |
|  | (0.00) |  | (0.00) |
| cov(e.European Social Citizenship Support2,e.Economic Pol. Orientation2) | 0.00^***^ | cov(e.Welfare Solidarity2,e.Economic Pol. Orientation2) | 0.00^***^ |
|  | (0.00) |  | (0.00) |
| cov(e.European Social Citizenship Support2,e.Immigration Support2) | 0.01^***^ | cov(e.Welfare Solidarity2,e.Immigration Support2) | 0.01^***^ |
|  | (0.00) |  | (0.00) |
| cov(e.European Social Citizenship Support2,e.Personal Income2) | -0.00 | cov(e.Welfare Solidarity2,e.Personal Income2) | -0.00 |
|  | (0.00) |  | (0.00) |
| cov(e.German Identification2,e.European Identification2) | 0.01^***^ | cov(e.German Identification2,e.European Identification2) | 0.01^***^ |
|  | (0.00) |  | (0.00) |
| cov(e.Economic Pol. Orientation2,e.Immigration Support2) | 0.00^**^ | cov(e.Economic Pol. Orientation2,e.Immigration Support2) | 0.00^***^ |
|  | (0.00) |  | (0.00) |
| cov(European Social Citizenship Support1,German Identification1) | 0.00 | cov(Welfare Solidarity1,German Identification1) | 0.01^***^ |
|  | (0.00) |  | (0.00) |
| cov(European Social Citizenship Support1,European Identification1) | 0.03^***^ | cov(Welfare Solidarity1,European Identification1) | 0.04^***^ |
|  | (0.00) |  | (0.00) |
| cov(European Social Citizenship Support1,Economic Pol. Orientation1) | 0.03^***^ | cov(Welfare Solidarity1,Economic Pol. Orientation1) | 0.03^***^ |
|  | (0.00) |  | (0.00) |
| cov(European Social Citizenship Support1,Immigration Support1) | 0.07^***^ | cov(Welfare Solidarity1,Immigration Support1) | 0.05^***^ |
|  | (0.00) |  | (0.00) |
| cov(European Social Citizenship Support1,Personal Income1) | 0.00 | cov(Welfare Solidarity1,Personal Income1) | 0.00 |
|  | (0.00) |  | (0.00) |
| cov(German Identification1,European Identification1) | 0.03^***^ | cov(German Identification1,European Identification1) | 0.03^***^ |
|  | (0.00) |  | (0.00) |
| cov(German Identification1,Economic Pol. Orientation1) | -0.00^**^ | cov(German Identification1,Economic Pol. Orientation1) | -0.00^**^ |
|  | (0.00) |  | (0.00) |
| cov(German Identification1,Immigration Support1) | 0.00 | cov(German Identification1,Immigration Support1) | 0.00 |
|  | (0.00) |  | (0.00) |
| cov(German Identification1,Personal Income1) | 0.00^**^ | cov(German Identification1,Personal Income1) | 0.00^**^ |
|  | (0.00) |  | (0.00) |
| cov(European Identification1,Economic Pol. Orientation1) | 0.01^***^ | cov(European Identification1,Economic Pol. Orientation1) | 0.01^***^ |
|  | (0.00) |  | (0.00) |
| cov(European Identification1,Immigration Support1) | 0.04^***^ | cov(European Identification1,Immigration Support1) | 0.04^***^ |
|  | (0.00) |  | (0.00) |
| cov(European Identification1,Personal Income1) | 0.00^***^ | cov(European Identification1,Personal Income1) | 0.00^***^ |
|  | (0.00) |  | (0.00) |
| cov(Economic Pol. Orientation1,Immigration Support1) | 0.03^***^ | cov(Economic Pol. Orientation1,Immigration Support1) | 0.03^***^ |
|  | (0.00) |  | (0.00) |
| cov(Economic Pol. Orientation1,Personal Income1) | -0.00^***^ | cov(Economic Pol. Orientation1,Personal Income1) | -0.00^***^ |
|  | (0.00) |  | (0.00) |
| cov(Immigration Support1,Personal Income1) | 0.00^*^ | cov(Immigration Support1,Personal Income1) | 0.00^*^ |
|  | (0.00) |  | (0.00) |
| Observations | 2006 | Observations | 2006 |
| RMSEA | 0.044 | RMSEA | 0.044 |
| CFI | 0.985 | CFI | 0.985 |
| TLI | 0.971 | TLI | 0.971 |
| CD | 0.979 | CD | 0.977 |
| chi2_ms(26) | 128.516 | chi2_ms(26) | 126.967 |

^*^ *p* < 0.05, ^**^ *p* < 0.01, ^***^ *p* < 0.001

## A.8.2 GLES

I estimate cross-lagged panel models of independent variables and both fiscal and territorial European solidarity in waves 11 and 22 of the GLES panel.

The main information from these models is summarised in Table 34 (see Table 35 for the full model): the causal pathway between the independent variables and fiscal and territorial European solidarity run primarily in the expected direction: The coefficient in the hypothesised direction is larger in all cases except for W11-German Identification and W11-Economic Pol. Orientation to Fiscal solidarity.

Table 34: Summary of Cross-Lagged Associations in GLES data

|  | **SEM 1** | |  | **SEM 2** | |
| --- | --- | --- | --- | --- | --- |
|  | **Hypothesised direction  W11-Variable to  W22-Fiscal Solidarity** | **Opposite direction  W11-Fiscal Solidarity to W22-Variable** |  | **Hypothesised direction  W11-Variable to  W22-Territorial Solidarity** | **Opposite direction  W11- Territorial Solidarity to W22-Variable** |
|  | b/se | b/se |  | b/se | b/se |
| W11-Fiscal Solidarity | 0.39^***^ | 0.39^***^ | W11-Territorial Solidarity | 0.34^***^ | 0.34^***^ |
|  | (0.01) | (0.01) |  | (0.01) | (0.01) |
| W11-German Identification | -0.02 | 0.03^**^ | German Identification | 0.02 | 0.01 |
|  | (0.01) | (0.01) |  | (0.01) | (0.01) |
| W11-European Identification | 0.12^***^ | 0.09^***^ | European Identification | 0.11^***^ | 0.03^*^ |
|  | (0.01) | (0.01) |  | (0.01) | (0.01) |
| W11-Economic Pol. Orientation | 0.05^***^ | 0.06^***^ | W11-Economic Pol. Orientation | 0.22^***^ | 0.07^***^ |
|  | (0.01) | (0.01) |  | (0.02) | (0.01) |
| W11-Transnational Pol. Orientation | 0.25^***^ | 0.08^***^ | W11-Transnational Pol. Orientation | 0.05^***^ | 0.03^***^ |
|  | (0.02) | (0.01) |  | (0.02) | (0.01) |
| W11-Personal Econ. Situation | -0.00 | 0.04^***^ | W11-Personal Econ. Situation | -0.01 | -0.01 |
|  | (0.01) | (0.01) |  | (0.01) | (0.01) |
| W11-General Econ. Situation | 0.05^***^ | 0.04^***^ | W11-General Econ. Situation | 0.01 | -0.01 |
|  | (0.01) | (0.01) |  | (0.02) | (0.01) |
| Constant | 0.06^***^ |  | Constant | 0.15^***^ |  |
|  | (0.01) |  |  | (0.02) |  |

Table 35: Full Result of Cross-Lagged Panel Models in GLES

| **SEM (1)** | | **SEM (2)** | |
| --- | --- | --- | --- |
|  | b/se |  | b/se |
| W22-Fiscal Solidarity |  | W22-Territorial Solidarity |  |
| W11-Fiscal Solidarity | 0.39^***^ | W11-Territorial Solidarity | 0.34^***^ |
|  | (0.01) |  | (0.01) |
| W11-German Identification | -0.02 | German Identification | 0.02 |
|  | (0.01) |  | (0.01) |
| W11-European Identification | 0.12^***^ | European Identification | 0.11^***^ |
|  | (0.01) |  | (0.01) |
| W11-Economic Pol. Orientation | 0.05^***^ | W11-Economic Pol. Orientation | 0.22^***^ |
|  | (0.01) |  | (0.02) |
| W11-Transnational Pol. Orientation | 0.25^***^ | W11-Transnational Pol. Orientation | 0.05^***^ |
|  | (0.02) |  | (0.02) |
| W11-Personal Econ. Situation | -0.00 | W11-Personal Econ. Situation | -0.01 |
|  | (0.01) |  | (0.01) |
| W11-General Econ. Situation | 0.05^***^ | W11-General Econ. Situation | 0.01 |
|  | (0.01) |  | (0.02) |
| Constant | 0.06^***^ | Constant | 0.15^***^ |
|  | (0.01) |  | (0.02) |
| German Identification_22 |  | German Identification_22 |  |
| W11-Fiscal Solidarity | 0.03^**^ | W11-Territorial Solidarity | 0.01 |
|  | (0.01) |  | (0.01) |
| German Identification | 0.66^***^ | German Identification | 0.65^***^ |
|  | (0.01) |  | (0.01) |
| European Identification | 0.08^***^ | European Identification | 0.10^***^ |
|  | (0.01) |  | (0.01) |
| Constant | 0.15^***^ | Constant | 0.16^***^ |
|  | (0.01) |  | (0.01) |
| European Identification_22 |  | European Identification_22 |  |
| W11-Fiscal Solidarity | 0.09^***^ | W11-Territorial Solidarity | 0.03^*^ |
|  | (0.01) |  | (0.01) |
| German Identification | 0.09^***^ | German Identification | 0.08^***^ |
|  | (0.01) |  | (0.01) |
| European Identification | 0.58^***^ | European Identification | 0.60^***^ |
|  | (0.01) |  | (0.01) |
| W11-Transnational Pol. Orientation | 0.19^***^ | W11-Transnational Pol. Orientation | 0.23^***^ |
|  | (0.01) |  | (0.01) |
| Constant | -0.01 | Constant | -0.01 |
|  | (0.01) |  | (0.01) |
| W22-Economic Orientation |  | W22-Economic Orientation |  |
| W11-Fiscal Solidarity | 0.06^***^ | W11-Territorial Solidarity | 0.07^***^ |
|  | (0.01) |  | (0.01) |
| W11-Economic Pol. Orientation | 0.63^***^ | W11-Economic Pol. Orientation | 0.63^***^ |
|  | (0.01) |  | (0.01) |
| W11-Personal Econ. Situation | -0.07^***^ | W11-Personal Econ. Situation | -0.07^***^ |
|  | (0.01) |  | (0.01) |
| Constant | 0.22^***^ | Constant | 0.20^***^ |
|  | (0.01) |  | (0.01) |
| W22-Transnational Orientation |  | W22-Transnational Orientation |  |
| W11-Fiscal Solidarity | 0.08^***^ | W11-Territorial Solidarity | 0.03^***^ |
|  | (0.01) |  | (0.01) |
| European Identification | 0.08^***^ | European Identification | 0.09^***^ |
|  | (0.01) |  | (0.01) |
| W11-Transnational Pol. Orientation | 0.70^***^ | W11-Transnational Pol. Orientation | 0.73^***^ |
|  | (0.01) |  | (0.01) |
| Constant | 0.06^***^ | Constant | 0.05^***^ |
|  | (0.00) |  | (0.00) |
| W22-Personal Econ. Situation |  | W22-Personal Econ. Situation |  |
| W11-Fiscal Solidarity | 0.04^***^ | W11-Territorial Solidarity | -0.01 |
|  | (0.01) |  | (0.01) |
| W11-Personal Econ. Situation | 0.62^***^ | W11-Personal Econ. Situation | 0.62^***^ |
|  | (0.01) |  | (0.01) |
| W11-General Econ. Situation | 0.08^***^ | W11-General Econ. Situation | 0.10^***^ |
|  | (0.01) |  | (0.01) |
| Constant | 0.13^***^ | Constant | 0.14^***^ |
|  | (0.01) |  | (0.01) |
| W22-General Econ. Situation |  | W22-General Econ. Situation |  |
| W11-Fiscal Solidarity | 0.04^***^ | W11-Territorial Solidarity | -0.01 |
|  | (0.01) |  | (0.01) |
| European Identification | 0.06^***^ | European Identification | 0.06^***^ |
|  | (0.01) |  | (0.01) |
| W11-Economic Pol. Orientation | 0.05^***^ | W11-Economic Pol. Orientation | 0.06^***^ |
|  | (0.01) |  | (0.01) |
| W11-Transnational Pol. Orientation | 0.11^***^ | W11-Transnational Pol. Orientation | 0.13^***^ |
|  | (0.01) |  | (0.01) |
| W11-Personal Econ. Situation | 0.09^***^ | W11-Personal Econ. Situation | 0.09^***^ |
|  | (0.01) |  | (0.01) |
| W11-General Econ. Situation | 0.39^***^ | W11-General Econ. Situation | 0.40^***^ |
|  | (0.01) |  | (0.01) |
| Constant | 0.06^***^ | Constant | 0.06^***^ |
|  | (0.01) |  | (0.01) |
| / |  | / |  |
| mean(Fiscal Solidarity_11) | 0.41^***^ | mean(Territorial Solidarity_11) | 0.62^***^ |
|  | (0.00) |  | (0.00) |
| mean(German Identification_11) | 0.73^***^ | mean(German Identification_11) | 0.73^***^ |
|  | (0.00) |  | (0.00) |
| mean(European Identification_11) | 0.53^***^ | mean(European Identification_11) | 0.53^***^ |
|  | (0.00) |  | (0.00) |
| mean(Economic Pol. Orientation_11) | 0.59^***^ | mean(Economic Pol. Orientation_11) | 0.59^***^ |
|  | (0.00) |  | (0.00) |
| mean(Transnational Orientation_11) | 0.41^***^ | mean(Transnational Orientation_11) | 0.41^***^ |
|  | (0.00) |  | (0.00) |
| mean(Personal Econ. Situation_11) | 0.60^***^ | mean(Personal Econ. Situation_11) | 0.60^***^ |
|  | (0.00) |  | (0.00) |
| mean(General Econ. Situation_11) | 0.62^***^ | mean(General Econ. Situation_11) | 0.62^***^ |
|  | (0.00) |  | (0.00) |
| var(e.Fiscal Solidarity_22) | 0.04^***^ | var(e.Territorial Solidarity_22) | 0.04^***^ |
|  | (0.00) |  | (0.00) |
| var(e.German Identification_22) | 0.04^***^ | var(e.German Identification_22) | 0.04^***^ |
|  | (0.00) |  | (0.00) |
| var(e.European Identification_22) | 0.04^***^ | var(e.European Identification_22) | 0.04^***^ |
|  | (0.00) |  | (0.00) |
| var(e.Economic Pol. Orientation_22) | 0.02^***^ | var(e.Economic Pol. Orientation_22) | 0.02^***^ |
|  | (0.00) |  | (0.00) |
| var(e.Transnational Orientation_22) | 0.02^***^ | var(e.Transnational Orientation_22) | 0.02^***^ |
|  | (0.00) |  | (0.00) |
| var(e.Personal Econ. Situation_22) | 0.03^***^ | var(e.Personal Econ. Situation_22) | 0.03^***^ |
|  | (0.00) |  | (0.00) |
| var(e.General Econ. Situation_22) | 0.03^***^ | var(e.General Econ. Situation_22) | 0.03^***^ |
|  | (0.00) |  | (0.00) |
| var(Fiscal Solidarity_11) | 0.07^***^ | var(Territorial Solidarity_11) | 0.06^***^ |
|  | (0.00) |  | (0.00) |
| var(German Identification_11) | 0.06^***^ | var(German Identification_11) | 0.06^***^ |
|  | (0.00) |  | (0.00) |
| var(European Identification_11) | 0.08^***^ | var(European Identification_11) | 0.08^***^ |
|  | (0.00) |  | (0.00) |
| var(Economic Pol. Orientation_11) | 0.03^***^ | var(Economic Pol. Orientation_11) | 0.03^***^ |
|  | (0.00) |  | (0.00) |
| var(Transnational Orientation_11) | 0.05^***^ | var(Transnational Orientation_11) | 0.05^***^ |
|  | (0.00) |  | (0.00) |
| var(Personal Econ. Situation_11) | 0.05^***^ | var(Personal Econ. Situation_11) | 0.05^***^ |
|  | (0.00) |  | (0.00) |
| var(General Econ. Situation_11) | 0.04^***^ | var(General Econ. Situation_11) | 0.04^***^ |
|  | (0.00) |  | (0.00) |
| cov(e.Fiscal Solidarity_22,e.German Identification_22) | 0.00^***^ | cov(e.Territorial Solidarity_22,e.German Identification_22) | 0.00^*^ |
|  | (0.00) |  | (0.00) |
| cov(e.Fiscal Solidarity_22,e.European Identification_22) | 0.01^***^ | cov(e.Territorial Solidarity_22,e.European Identification_22) | 0.00^***^ |
|  | (0.00) |  | (0.00) |
| cov(e.Fiscal Solidarity_22,e.Economic Pol. Orientation_22) | 0.00^***^ | cov(e.Territorial Solidarity_22,e.Economic Pol. Orientation_22) | 0.01^***^ |
|  | (0.00) |  | (0.00) |
| cov(e.Fiscal Solidarity_22,e.Transnational Orientation_22) | 0.01^***^ | cov(e.Territorial Solidarity_22,e.Transnational Orientation_22) | 0.00^***^ |
|  | (0.00) |  | (0.00) |
| cov(e.Fiscal Solidarity_22,e.Personal Econ. Situation_22) | 0.00^***^ | cov(e.Territorial Solidarity_22,e.Personal Econ. Situation_22) | 0.00 |
|  | (0.00) |  | (0.00) |
| cov(e.Fiscal Solidarity_22,e.General Econ. Situation_22) | 0.00^***^ | cov(e.Territorial Solidarity_22,e.General Econ. Situation_22) | 0.00 |
|  | (0.00) |  | (0.00) |
| cov(e.German Identification_22,e.European Identification_22) | 0.01^***^ | cov(e.German Identification_22,e.European Identification_22) | 0.01^***^ |
|  | (0.00) |  | (0.00) |
| cov(e.German Identification_22,e.Transnational Orientation_22) | 0.00^***^ | cov(e.German Identification_22,e.Transnational Orientation_22) | 0.00^***^ |
|  | (0.00) |  | (0.00) |
| cov(e.European Identification_22,e.Transnational Orientation_22) | 0.01^***^ | cov(e.European Identification_22,e.Transnational Orientation_22) | 0.01^***^ |
|  | (0.00) |  | (0.00) |
| cov(e.Economic Pol. Orientation_22,e.Transnational Orientation_22) | 0.00^***^ | cov(e.Economic Pol. Orientation_22,e.Transnational Orientation_22) | 0.00^***^ |
|  | (0.00) |  | (0.00) |
| cov(e.Personal Econ. Situation_22,e.General Econ. Situation_22) | 0.01^***^ | cov(e.Personal Econ. Situation_22,e.General Econ. Situation_22) | 0.01^***^ |
|  | (0.00) |  | (0.00) |
| cov(Fiscal Solidarity_11,German Identification_11) | -0.00^***^ | cov(Territorial Solidarity_11,German Identification_11) | 0.00^**^ |
|  | (0.00) |  | (0.00) |
| cov(Fiscal Solidarity_11,European Identification_11) | 0.03^***^ | cov(Territorial Solidarity_11,European Identification_11) | 0.02^***^ |
|  | (0.00) |  | (0.00) |
| cov(Fiscal Solidarity_11,Economic Pol. Orientation_11) | 0.02^***^ | cov(Territorial Solidarity_11,Economic Pol. Orientation_11) | 0.02^***^ |
|  | (0.00) |  | (0.00) |
| cov(Fiscal Solidarity_11,Transnational Orientation_11) | 0.03^***^ | cov(Territorial Solidarity_11,Transnational Orientation_11) | 0.02^***^ |
|  | (0.00) |  | (0.00) |
| cov(Fiscal Solidarity_11,Personal Econ. Situation_11) | 0.01^***^ | cov(Territorial Solidarity_11,Personal Econ. Situation_11) | 0.00^***^ |
|  | (0.00) |  | (0.00) |
| cov(Fiscal Solidarity_11,General Econ. Situation_11) | 0.01^***^ | cov(Territorial Solidarity_11,General Econ. Situation_11) | 0.01^***^ |
|  | (0.00) |  | (0.00) |
| cov(German Identification_11,European Identification_11) | 0.02^***^ | cov(German Identification_11,European Identification_11) | 0.02^***^ |
|  | (0.00) |  | (0.00) |
| cov(German Identification_11,Economic Pol. Orientation_11) | -0.00^**^ | cov(German Identification_11,Economic Pol. Orientation_11) | -0.00^**^ |
|  | (0.00) |  | (0.00) |
| cov(German Identification_11,Transnational Orientation_11) | -0.01^***^ | cov(German Identification_11,Transnational Orientation_11) | -0.01^***^ |
|  | (0.00) |  | (0.00) |
| cov(German Identification_11,Personal Econ. Situation_11) | 0.01^***^ | cov(German Identification_11,Personal Econ. Situation_11) | 0.01^***^ |
|  | (0.00) |  | (0.00) |
| cov(German Identification_11,General Econ. Situation_11) | 0.01^***^ | cov(German Identification_11,General Econ. Situation_11) | 0.01^***^ |
|  | (0.00) |  | (0.00) |
| cov(European Identification_11,Economic Pol. Orientation_11) | 0.01^***^ | cov(European Identification_11,Economic Pol. Orientation_11) | 0.01^***^ |
|  | (0.00) |  | (0.00) |
| cov(European Identification_11,Transnational Orientation_11) | 0.03^***^ | cov(European Identification_11,Transnational Orientation_11) | 0.03^***^ |
|  | (0.00) |  | (0.00) |
| cov(European Identification_11,Personal Econ. Situation_11) | 0.01^***^ | cov(European Identification_11,Personal Econ. Situation_11) | 0.01^***^ |
|  | (0.00) |  | (0.00) |
| cov(European Identification_11,General Econ. Situation_11) | 0.02^***^ | cov(European Identification_11,General Econ. Situation_11) | 0.02^***^ |
|  | (0.00) |  | (0.00) |
| cov(Economic Pol. Orientation_11,Transnational Orientation_11) | 0.01^***^ | cov(Economic Pol. Orientation_11,Transnational Orientation_11) | 0.01^***^ |
|  | (0.00) |  | (0.00) |
| cov(Economic Pol. Orientation_11,Personal Econ. Situation_11) | -0.01^***^ | cov(Economic Pol. Orientation_11,Personal Econ. Situation_11) | -0.01^***^ |
|  | (0.00) |  | (0.00) |
| cov(Economic Pol. Orientation_11,General Econ. Situation_11) | 0.00^***^ | cov(Economic Pol. Orientation_11,General Econ. Situation_11) | 0.00^***^ |
|  | (0.00) |  | (0.00) |
| cov(Transnational Orientation_11,Personal Econ. Situation_11) | 0.01^***^ | cov(Transnational Orientation_11,Personal Econ. Situation_11) | 0.01^***^ |
|  | (0.00) |  | (0.00) |
| cov(Transnational Orientation_11,General Econ. Situation_11) | 0.01^***^ | cov(Transnational Orientation_11,General Econ. Situation_11) | 0.01^***^ |
|  | (0.00) |  | (0.00) |
| cov(Personal Econ. Situation_11,General Econ. Situation_11) | 0.02^***^ | cov(Personal Econ. Situation_11,General Econ. Situation_11) | 0.02^***^ |
|  | (0.00) |  | (0.00) |
| Observations | 9502 | Observations | 9502 |
| RMSEA | 0.048 | RMSEA | 0.051 |
| CFI | 0.979 | CFI | 0.974 |
| TLI | 0.950 | TLI | 0.940 |
| CD | 0.966 | CD | 0.965 |
| chi2_ms(30) | 685.605 | chi2_ms(30) | 770.926 |

^*^ *p* < 0.05, ^**^ *p* < 0.01, ^***^ *p* < 0.001

# References

Andreß, H.-J., Golsch, K., & Schmidt, A. W. (2013). *Applied Panel Data Analysis for Economic and Social Surveys*. Springer. https://doi.org/10.1007/978-3-642-32914-2

GLES. (2022). *GLES Panel 2022, Wave 22* (Version 1.0.0) [Dataset]. GESIS. https://doi.org/10.4232/1.13970

GLES. (2023). *GLES Panel 2016-2021, Waves 1-21* (No. ZA6838; Version 6.0.0) [Dataset]. GESIS. https://doi.org/10.4232/1.14114

Keele, L., & Kelly, N. J. (2006). Dynamic Models for Dynamic Theories: The Ins and Outs of Lagged Dependent Variables. *Political Analysis*, *14*(2), 186–205. https://doi.org/10.1093/pan/mpj006

Scott Long, J. (2014). Regression models for nominal and ordinal outcomes. In H. Best & C. Wolf (Eds.), *The SAGE Handbook of Regression Analysis and Causal Inference* (pp. 173–203). SAGE Publications Ltd. https://doi.org/10.4135/9781446288146

Vaisey, S., & Miles, A. (2017). What You Can—And Can’t—Do With Three-Wave Panel Data. *Sociological Methods & Research*, *46*(1), 44–67. https://doi.org/10.1177/0049124114547769

Wilkins, A. S. (2018). To Lag or Not to Lag?: Re-Evaluating the Use of Lagged Dependent Variables in Regression Analysis. *Political Science Research and Methods*, *6*(2), 393–411. https://doi.org/10.1017/psrm.2017.4
